# Supplementary material for: Restricting datasets to classifiable samples augments discovery of immune disease biomarkers
Source: Nat Commun. 2024 Jun 26;15:5417. doi: 10.1038/s41467-024-49094-3 (PMC11208602; doi:10.1038/s41467-024-49094-3)
Supplement: Supplementary file 1 — Supplementary Information [file 41467_2024_49094_MOESM1_ESM.pdf]

–SUPPLEMENTARY INFORMATION–

Restricting datasets to classifiable samples augments discovery of immune disease biomarkers

Gunther Glehr[1], Paloma Riquelme[1], Katharina Kronenberg[1], Robert Lohmayer[2], Víctor J. López-Madrona[3], Michael Kapinsky[4], Hans J. Schlitt[1], Edward K. Geissler[1], Rainer Spang[5], Sebastian Haferkamp[6], and James A. Hutchinson[1,*]

[1]Department of Surgery, University Hospital Regensburg, Regensburg, Germany
[2]Algorithmic Bioinformatics Research Group, Leibniz Institute for Immunotherapy, Regensburg, Germany
[3]Aix Marseille Univ, INSERM, Institut de Neurosciences des Systèmes, Marseille, France
[4]Beckman Coulter Life Sciences GmbH, Krefeld, Germany
[5]Department of Statistical Bioinformatics, University of Regensburg, Regensburg, Germany
[6]Department of Dermatology, University Hospital Regensburg, Regensburg, Germany
[*]Corresponding author: James A. Hutchinson, james.hutchinson@ukr.de

Supplementary Notes

Supplementary Note 1: Class sizes and discriminatory value **S3**

Supplementary Note 2: Website for data simulation **S6**

Supplementary Note 3: ROC definition and probabilistic interpretation **S30**
3.1 Definition of the ROC . . . S30
3.2 The area under the ROC curve (AUC) . . . S33

Supplementary Note 4: Restriction method **S34**
4.1 Partial area under the ROC curve (pAUC) . . . S34
4.2 Two-way partial area under the ROC curve . . . S35
4.2.1 AUC_{high} . . . S37
4.2.2 AUC_{low} . . . S38
4.3 Restricted sample space . . . S39
4.3.1 ROC for restricted sample space . . . S39
4.3.2 AUC for restricted sample space . . . S40
4.3.3 Restricted AUC in terms of true and false positive rates . . . S41
4.4 Restriction . . . S42
4.4.1 $\text{rAUC}_{high}(r)$ . . . S42
4.4.2 $\text{rAUC}_{low}(r)$ . . . S42

References **S50**

Supplementary Figures

1 Class sizes do not determine the informative value of disease biomarkers. S3
2 FlowSOM clustering identifies TCR$\gamma\delta^{+}$ cells as a biomarker for hepatitis. S7
3 Restriction applied to count distributions S9
4 Swapped positive and negative class restriction. S10
5 Schematic gating scheme for one real and three synthetic samples. S11
6 The restricted standardized AUC is biased. S12
7 Restriction applied to randomly labelled data S13
8 Significant biomarkers according to classical and restricted analysis predicting colitis. . . . S14
9 Restriction applied to proteomics - 1 . . . . S15
10 Restriction applied to proteomics - 2 . . . . S17
11 Restriction applied to microbiomics S19
12 Restriction applied to mass cytometry S21
13 Restriction applied to transcriptomics S23
14 Multivariate restriction applied to transcriptomics S26
15 Multivariate restriction applied to transcriptomics with random train/test splits S28
16 ROC curve with explicit threshold notation S32
17 Two-way partial area under the ROC curve. S35
18 ROC curves showing two-way partial AUCs corresponding to $marker^{HIGH}$ and $marker^{LOW}$ samples used in our restriction method. S37
19 Densities, ROC curves, restricted AUCs and restricted standardized AUCs. S43
20 Pre-processing of flow cytometry data. S44
21 Estimating cell population parameters as a basis for synthesizing realistic flow cytometry data.S45
22 Dirichlet probability density examples for K=3. . . . . S47
23 Procedure for generating realistic flow cytometry data. S49

Supplementary Tables

1 Patient cohort characteristics of training and validation set. S29
2 Parameters, means, variances and covariances for probability densities shown in Supplementary Figure 22. . . . . S48

Supplementary Note 1: Class sizes and discriminatory value

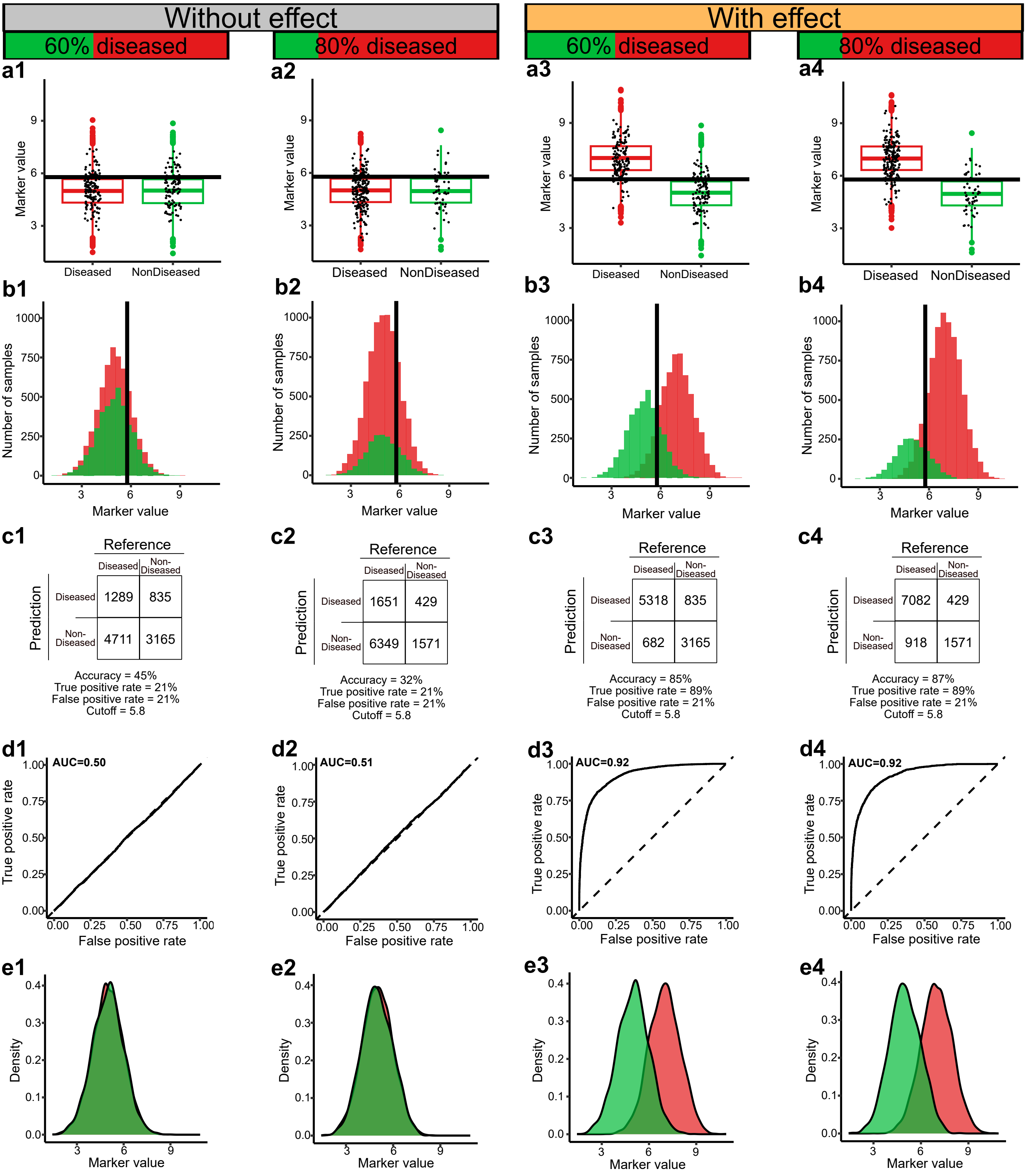

Supplementary Figure 1 *Class sizes do not determine the informative value of disease biomarkers.*

In this supplementary note, we explain the relationship between the discriminatory value of disease biomarkers, class sizes and correct classification rates. Most readers intuitively appreciate that a test's accuracy (also called the correct classification rate) depends upon the relative number of cases in each class. However, a marker's discriminatory value is independent of class size. Figure 1 explains this critical distinction through four illustrated examples, arranged in columns (1 - 4) each consisting of 5 panels (a - e).

The vertically arranged panels depict the same datasets treated in five different ways:

- **(a)** Boxplot of the biomarker values for both diseased (red) and non-diseased (green) samples. 250 randomly selected samples represented as individual points. The boxplot provides a visual summary of the distribution of the biomarker values. An arbitrary discriminatory cutoff is displayed at a biomarker value of 5.8.
- **(b)** Histogram of biomarker values for diseased (red) and non-diseased (green) samples. The histogram illustrates the distribution of cases with respect to biomarker values in each class. The same arbitrary discriminatory cutoff is displayed at a biomarker value of 5.8.
- **(c)** Confusion matrix summarizing the predicted class according to the arbitrary biomarker value cutoff value of 5.8. This table shows the number of true positives (TP), true negatives (TN), false positives (FP), and false negatives (FP). From these numbers, we can calculate the true positive rate (TPR), false positive rate (FPR) and accuracy. Hence, the confusion matrix helps to evaluate the performance of a biomarker in predicting disease status for one specific cutoff value.
- **(d)** Receiver operating characteristic (ROC) curves are graphical representations of the performance of a biomarker in distinguishing between diseased and non-diseased samples. ROC curves plot the true positive rate (sensitivity) against the false positive rate (1-specificity) calculated for all biomarker values in the dataset. As described in Figure 1, the area under the ROC (AUC) is a measure of a marker's discriminatory power.
- **(e)** Densities for diseased (red) and non-diseased samples. Imagine a histogram divided by the total number of samples in the respective class.

Let us first consider columns 1 and 2, which represent a biomarker with no discriminatory value:

- **(a)** Regardless of the number of diseased (red: column 1, n=6000; column 2, n=8000) or non-diseased (green: column 1, n=4000; column 2, n=2000) samples in each class, the boxplots shown in (a1) and (a2) are almost indistinguishable.
- **(b)** In contrast, the histograms in (b1) and (b2) clearly show more diseased samples in column 2.
- **(c)** By definition, no biomarker value is useful for discriminating between the two classes. Our arbitrary choice of 5.8 as a cutoff leads to confusion tables (c1) and (c2). The accuracy of this test decreases from (c1; 45%) to (c2; 32%) because more samples happen to fall below the cutoff. Importantly, TPR (21%) and FPR (21%) are identical irrespective of class size.
- **(d)** The ROC curves in (d1) and (d2) plot TPR against FPR for these samples. Because TPR and FPR are not affected by class size, the ROC curves and their area under the curve are essentially identical (d1: AUC = 0.50; d2: AUC = 0.51). Importantly, our interpretation of these curves as showing a biomarker with no discriminatory value is not affected by the different class sizes in (d1) and (d2).
- **(e)** The density plots shown in (e1) and (e2) help us to understand why our hypothetical biomarker has no discriminatory power. By normalizing the class sizes, we see the biomarker distributions in the diseased (red) and non-diseased (green) classes are essentially identically shaped and completely overlapping. ROC curves are based on densities, not absolute numbers of cases.

Let us now consider columns 3 and 4, which represent a biomarker with a high discriminatory value:

- **(a)** Regardless of the number of diseased (red: column 3, n=6000; column 4, n=8000) or non-diseased (green: column 3, n=4000; column 4, n=2000) samples in each class, the boxplots shown in (a1) and (a2) are almost indistinguishable.

(b) In contrast, the histograms in (b3) and (b4) clearly show more diseased samples in column 4.

(c) Our arbitrary choice of 5.8 as a cutoff leads to confusion tables (c3) and (c4). The accuracy of this test increases slightly from (c3; 85%) to (c4; 87%) owing to the relative differences in class sizes. Importantly, TPR (89%) and FPR (21%) are identical irrespective of class size.

(d) The ROC curves in (d3) and (d4) plot TPR against FPR for these samples. Because TPR and FPR are not affected by class size, the ROC curves are essentially identical (d3: AUC = 0.92; d4: AUC = 0.92). Importantly, our interpretation of these curves as showing a biomarker with high discriminatory value is not affected by the different class sizes in (d3) and (d4).

(e) The density plots shown in (e1) and (e2) help us to understand why our hypothetical biomarker has high discriminatory power. By normalizing the class sizes, we see the biomarker distributions in the diseased (red) and non-diseased (green) classes are essentially identically shaped but only have a small overlap.

In summary, class sizes may affect the accuracy of a test, but not its TPR or FPR. Hence, the discriminatory value of a marker, which is a function of TPR and FPR, is not determined by class sizes. Densities are a class size-independent way of visualising the expression of a disease biomarker within a patient class. Because densities are directly relevant to a marker's discriminatory value, we present plots of densities throughout this article.

Supplementary Note 2: Website

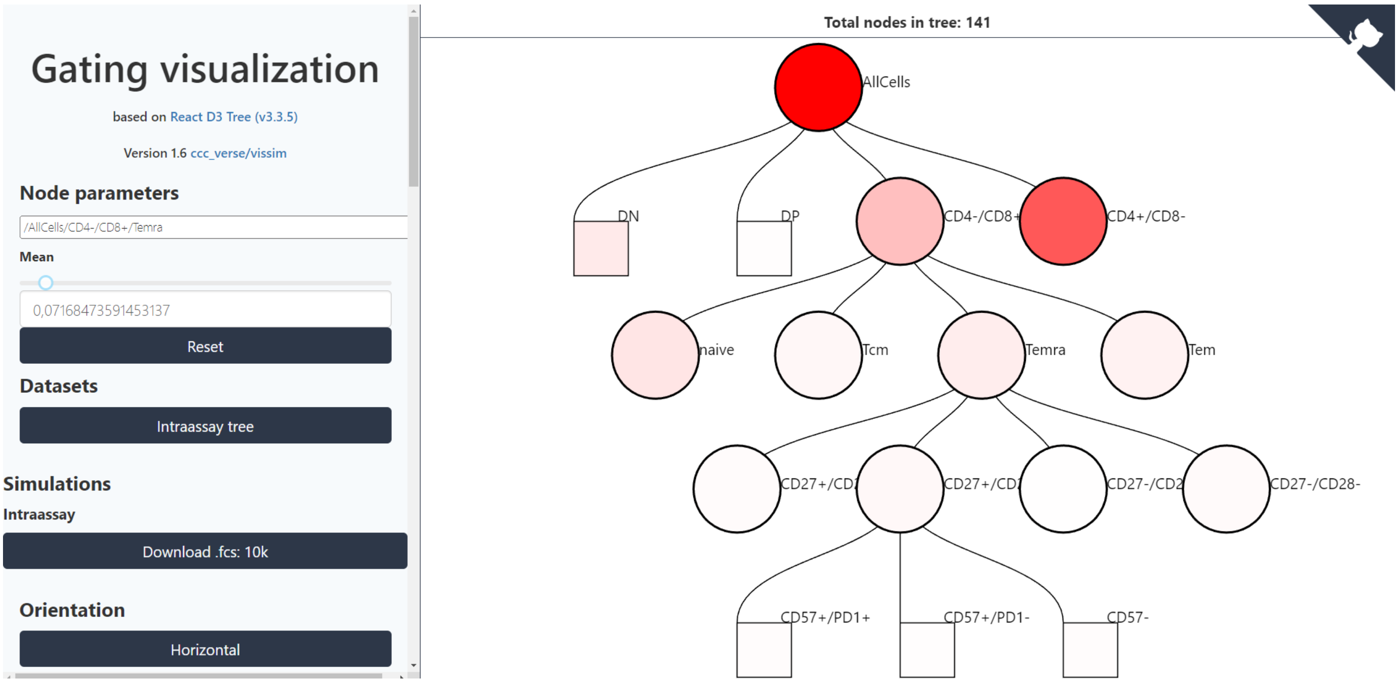

Supplementary Website 1 *vissim.gunthergl.com: Interactive gating tree to visualize our gating tree and to allow simulation of own flow cytometry datasets.*

A hierarchical gating tree used to classify peripheral blood leucocytes stained for T cell subset cell cell antigens. Within this hierarchy, intermediate gates (circles) define the cells that are categorized into end nodes by leaf gates (rectangles). Typical frequencies of cells in all gates were estimated from 48 samples, which represented 6 replicate stainings from 8 healthy donors. Gates are coloured according to the percentage of all cells they encompass. We pre-processed the data by pre-filtering for CD45$^+$ cells and show further gates in the figure. We filter for CD3$^+$ T cells and subsequently split into quadrants according to CD4 at 0.19 and CD8 at 0.2 into double negatives (DN), double positives (DP), CD4$^+$/CD8$^-$ and CD4$^-$/CD8$^+$. Only CD4$^+$/CD8$^-$ and CD4$^-$/CD8$^+$ gates are split into quadrants according to CCR7 at 0.24 and CD45RA at 0.12 into TEM (-,-), TEMRA (-,+), TCM (+,-) and Tnaive (+,+). Every one of those quadrants is again split into quadrants according to CD27 at 0.36 and CD28 at 0.30. Finally, every one of those quadrants is split into CD57$^-$ (CD57<=0.23), CD57$^+$/PD1$^+$ (CD57>0.23 and PD1>0.52) and CD57$^+$/PD1$^-$ (CD57>0.23 and PD1<=0.52) cells. Therefore the interactive tree has 141 nodes. Selecting any node shows the node mean corresponding to its expected value of the Dirichlet distribution. The mean can be changed with the slider, therefore we can directly change the underlying distribution. Finally, a .fcs file containing 10,000 cells can be downloaded.

Supplementary Figure 2

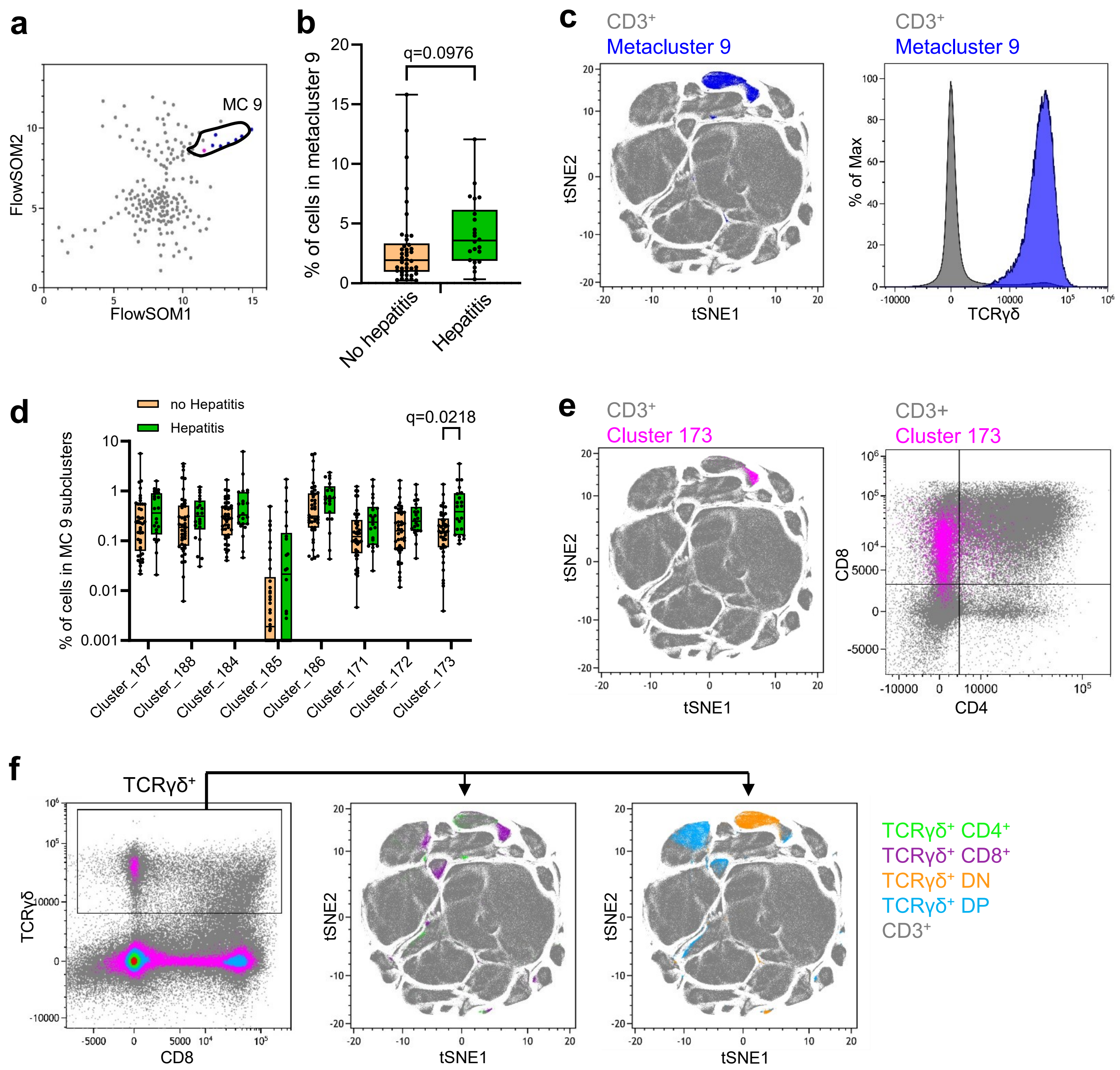

Supplementary Figure 2 *part 1/2. FlowSOM clustering results in TCR$\gamma\delta^+$ cells as a biomarker for hepatitis.* **(a)** PBMC from 64 patients were stimulated for 4 hours and stained for cell surface antigen and intracellular cytokines. FlowSOM analysis was performed on CD3$^+$ cells with 9 metaclusters and 196 clusters. Metacluster 9 coloured blue, cluster 173 coloured pink (n=42 no hepatitis, n=22 hepatitis). **(b)** There was a higher abundance of cells in metacluster 9 in patients who subsequently developed hepatitis, tested with the Mann-Whitney U test. **(c)** Metacluster 9 corresponded to a population of TCR$\gamma\delta^+$ cells. **(d)** The proportion of cells in cluster 173 was higher in hepatitis patients. We applied multiple Mann-Whitney U tests to compare the abundance of cells in each cluster between patients with and without hepatitis. P-values were adjusted for multiple testing using the Benjamini-Hochberg procedure. **(e)** Cluster 173 included mostly CD8$^+$ TCR$\gamma\delta^+$ cells. **(f)** TCR$\gamma\delta^+$ cells are distributed in different areas of the viSNE plot; metacluster 9 was mainly formed by CD8$^+$ and double negative (DN) T cells.

g

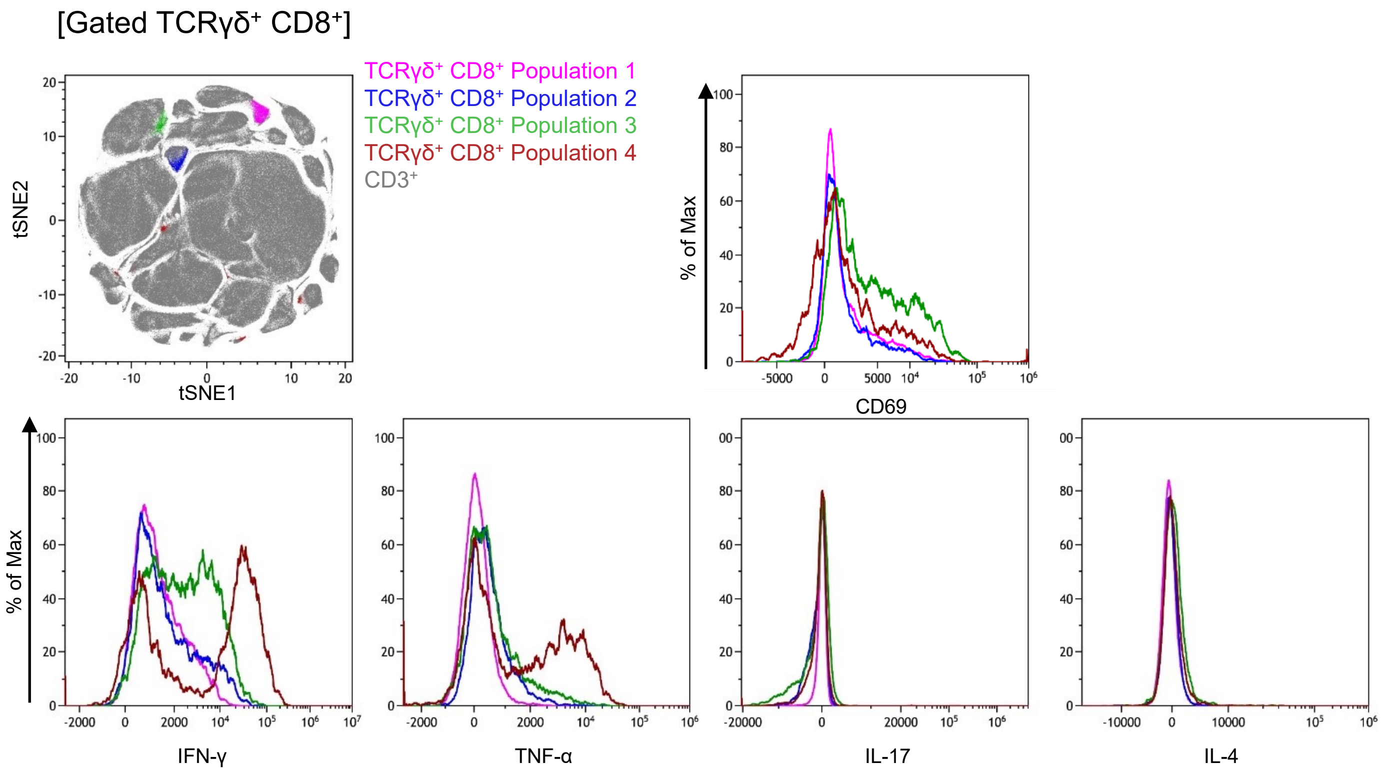

h

488 nm	561 nm				638 nm			405 nm				808 nm
525/40	585/42	610/20	710/50	763/43	660/10	712/25	763/43	450/45	525/40	610/20	763/43	885/40
**AF488**	**PE**	**AF594**	**PC5.5**	**PC7**	**AF647**	**AF700**	**AFire750**	**BV421**	**BV510**	BV605	**BV785**	Viability

Antigen	TCR vα7.2	IL-17	CD8	TCRγδ	TCR-Vα24	IL-4	CD4	CD3	IFNγ	TNFα	CD69	CD56	Live-Dead
Clone	3C10	BL168	RPA-T8	IMMU510	6B11	MP4-25D2	SK	UCHT1	B27	MAb11	FN50	5.1H11	
Isotype	mIgG1	mIgG1	mIgG1	mIgG1	mIgG1	rIgG1	mIgG1	mIgG1	mIgG1	mIgG1	mIgG1	mIgG1	
Fluorochrome	FITC	PE	AF594	PC5.5	PC7	APC	AF700	APC-A750	BV421	BV510	BV605	BV785	ViaKr-808
Vol. (µl)	3	2	3	6	3	1	3	5	2	2	5	5	2
Supplier	Biolegend	BioLegend	BioLegend	BC	BioLegend	BioLegend	BioLegend	BC	BioLegend	BioLegend	BioLegend	BioLegend	BC
Cat.#	351703	512306	301056	A99021	342911	500812	344621	A94680	506538	502950	310937	362549	C36628

Supplementary Figure 2 *part 2/2. FlowSOM clustering identifies TCR$\gamma\delta^+$ cells as a biomarker for hepatitis.* **(g)** TCR$\gamma\delta^+$ CD8$^+$ can be subdivided into 4 populations. Cluster 173 corresponds to Population 1, characterized by low CD69 expression and low cytokine production. **(h)** Staining panel used to analyze T cell populations in this experiment.

Supplementary Figure 3

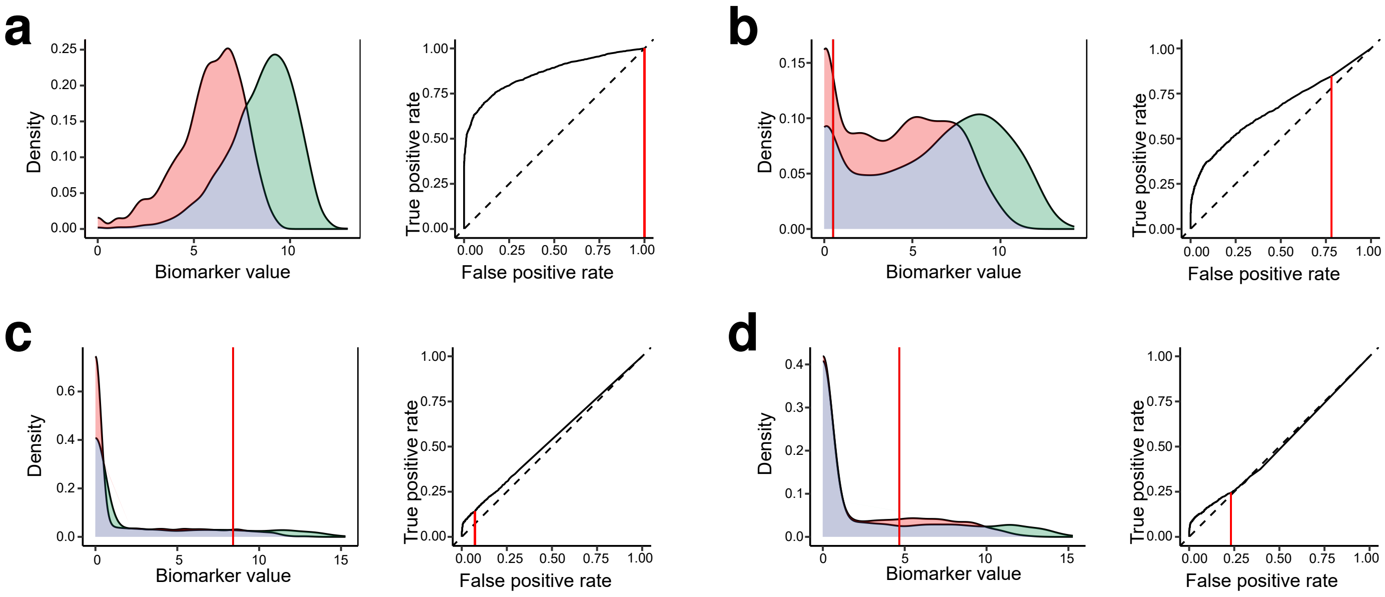

Supplementary Figure 3 *Restriction applied to count distributions*

Count distributions are usually modelled as coming from a negative binomial distribution. The negative binomial distribution $(NB(\mu, \phi))$ is parametrized by its mean μ and the dispersion ϕ. We show four examples of two classes from differently parametrized negative binomial distributions. The distributions are shown after $\log_2(\text{simulated counts} + 1)$ transformation. Note that our method would find the same informative range with or without log transformation because it is based on the ROC curve.

Each subfigure presents $\log_2$ transformed densities and their corresponding ROC curves. The method-selected restriction value is emphasized as a vertical red line. For both the positive and negative classes, 2500 samples were simulated. A $\log_2$-fold change of 2.5 was used as the difference between the two classes. We chose a mean count of 100 $(\log_2(100 + 1) = 6.66)$ for the negative population and a mean count of 625 $(\log_2(625 + 1) = 9.29)$ for the positive population.

(a) Simulated example of positive $(NB(9.29, 1))$ and negative $(NB(6.66, 1))$ samples are shown. Restriction identifies the full range as informative. **(b)** Simulated example of positive $(NB(9.29, .25))$ and negative $(NB(6.66, .25))$ samples are shown. Restriction excludes samples with zero counts. **(c)** Simulated example of positive $(NB(9.29, .05))$ and negative $(NB(6.66, .05))$ samples are shown. Restriction identifies the informative range as samples with more than $2^{8.403} \approx 338$ counts. **(d)** Simulated example of positive $(NB(9.29, .05))$ and negative $(NB(6.66, .07))$ samples are shown. Restriction identifies the informative range as samples with more than $2^{4.67} \approx 25$ counts.

Supplementary Figure 4

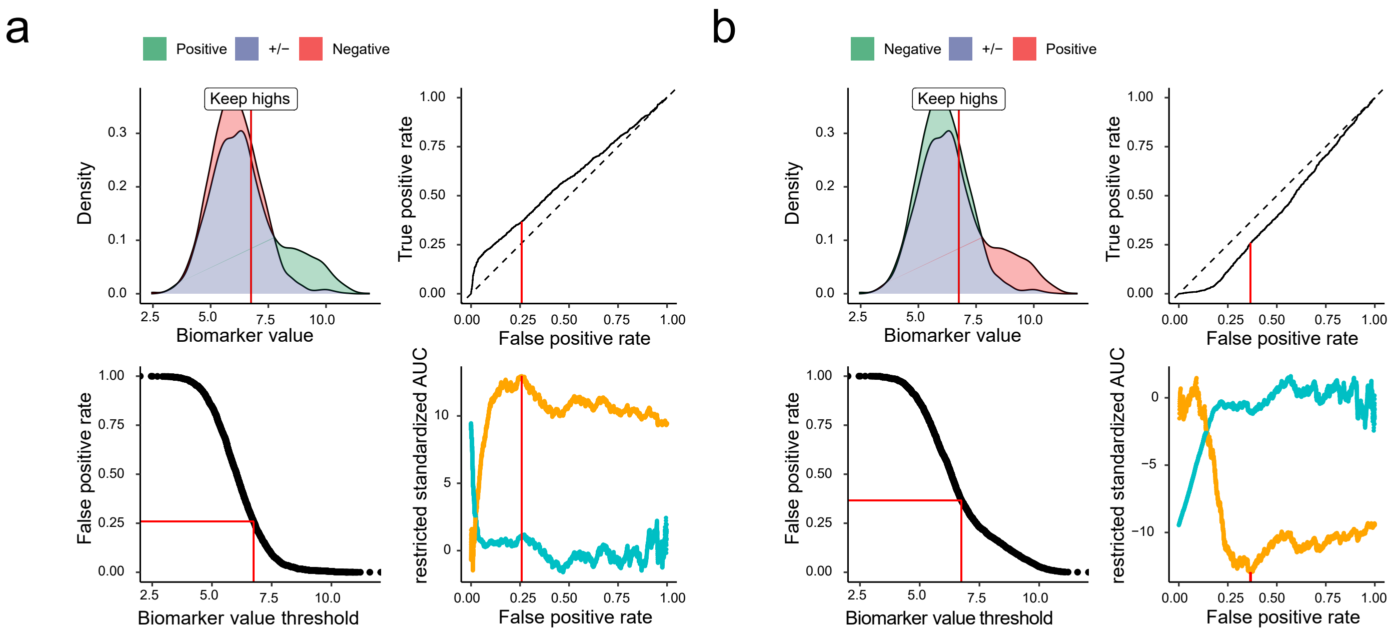

Supplementary Figure 4 *Swapped positive and negative class restriction.*
Exchanging the labels of the positive and negative classes leads to an inversion of the ROC curve, but does not affect the optimal restriction. **(a)** The top-left panel shows the distribution of 2500 positive and 2500 negative samples where 20% of all positive and 2% of all negative samples are different $\mathcal{N}(9,1)$ from the majority population $\mathcal{N}(6,1)$. The optimal restriction is indicated with a red line (value = 6.8) and marker^{HIGH} samples are kept. The top-right panel shows the complete ROC curve with the same restriction indicated at FPR = 0.258. The bottom-left panel relates FPR to biomarker values for all samples. The bottom-right panel shows the restricted standardized AUC (rzAUC) for every possible restriction calculated for marker^{HIGH} and marker^{LOW} samples. The optimal restriction is indicated by a red line. **(b)** This hypothetical example is identical to that shown in (a) except that the positive and negative class labels were switched when calculating the ROC curve. The top-left panel shows the distribution of 2500 negative and 2500 positive samples where 20% of all negative and 2% of all positive samples are different $\mathcal{N}(9,1)$ from the majority population $\mathcal{N}(6,1)$. The optimal restriction is indicated with a red line (value = 6.8) and marker^{HIGH} samples are kept which is identical to (a). The top-right panel shows the complete ROC curve with its restriction at FPR = 0.366. The bottom-left panel relates FPR to biomarker values for all samples. The bottom-right panel shows the restricted standardized AUC (rzAUC) for every possible restriction calculated for marker^{HIGH} and marker^{LOW} samples. The optimal restriction is indicated by a red line.

Supplementary Figure 5

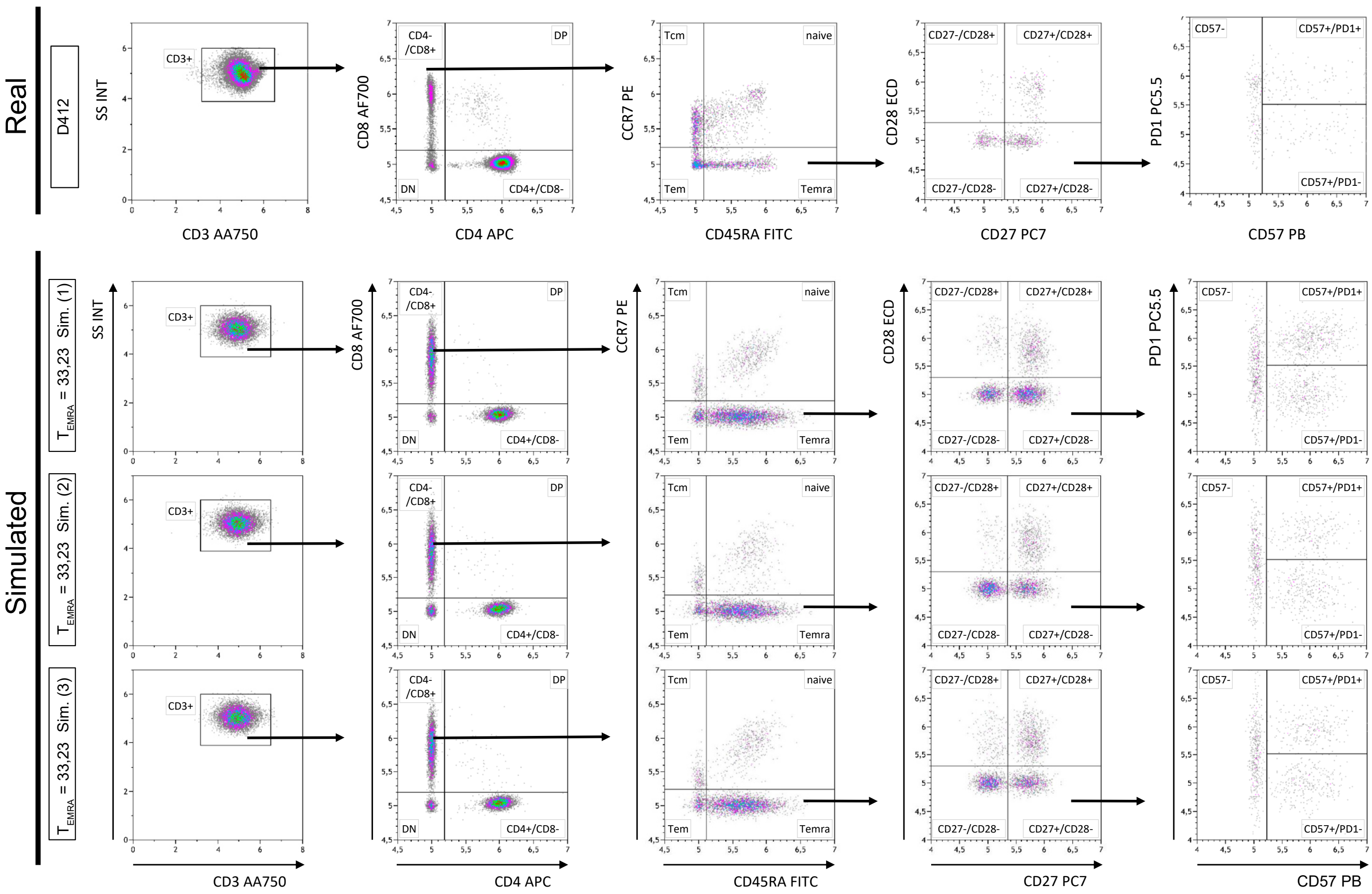

Supplementary Figure 5 *Schematic gating scheme for one real and three synthetic samples.*

In this paper, our gating schema for the DURAClone IM T Cell Subsets Tube gates 1) $CD3^+$ cells, 2) splits them into quadrants according to CD4 and CD8, 3) subsequently splits $CD4^+/CD8^-$ and $CD4^-/CD8^+$ into quadrants according to CD45RA and CCR7, 4) subsequently splits every quadrant into quadrants according to CD27 and CD28, 5) subsequently splits every quadrant into $CD57^-$, $CD57^+/PD1^+$ and $CD57^+/PD1^-$. The first row shows this gating for sample D142. The next three rows show the gating for three simulated samples where $CD8^+$ T_{EMRA} were increased to have a mean of 33.23% in contrast to a baseline mean of 7.17% in 8 donors with 6 replicates each.

Supplementary Figure 6

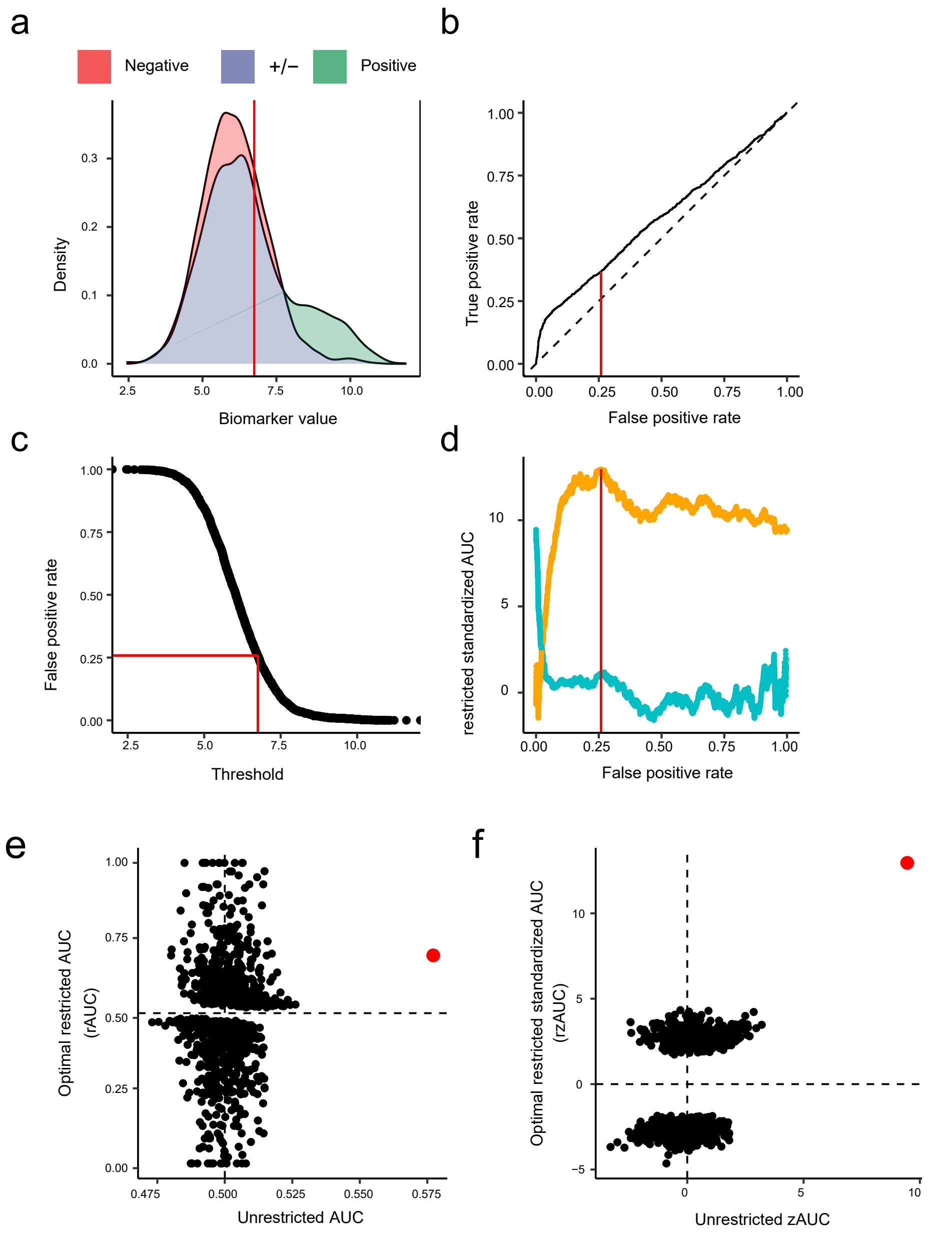

Supplementary Figure 6 *The restricted standardized AUC is biased.*

Although the AUC of the complete ROC curve is unbiased, the optimized rAUC and rzAUC are biased metrics; therefore, to compare the discriminatory performance of different biomarkers, we introduce permutation p-values. **(a)** A simulated distribution of 2500 positive and 2500 negative samples, where 20% of all positive and 2% of all negative samples are different $\mathcal{N}(9,1)$ from the majority population $\mathcal{N}(6,1)$. The optimal restriction (value = 6.8) is indicated by a red line. **(b)** The corresponding complete ROC curve for all samples with the optimal restriction (FPR = 0.258) is indicated by a red line. **(c)** A plot of biomarker values against FPR calculated for all samples. The optimal restriction is indicated by red lines. **(d)** The restricted standardized AUC (rzAUC) calculated for marker^{HIGH} (orange) and marker^{LOW} (blue) samples calculated for all possible restrictions. The optimal restriction is indicated by a red line. **(e)** Plot relating the AUC of the complete ROC curve to the rAUC for 10000 permutations of the positive- and negative-class labels. The red point represents the observed value for the unpermuted (i.e. correctly labelled) data. **(f)** Plot relating the standardized AUC of the complete ROC curve (zAUC) to the rzAUC for 10000 permutations of the positive- and negative-class labels. The red point represents the observed value for the unpermuted data.

Supplementary Figure 7

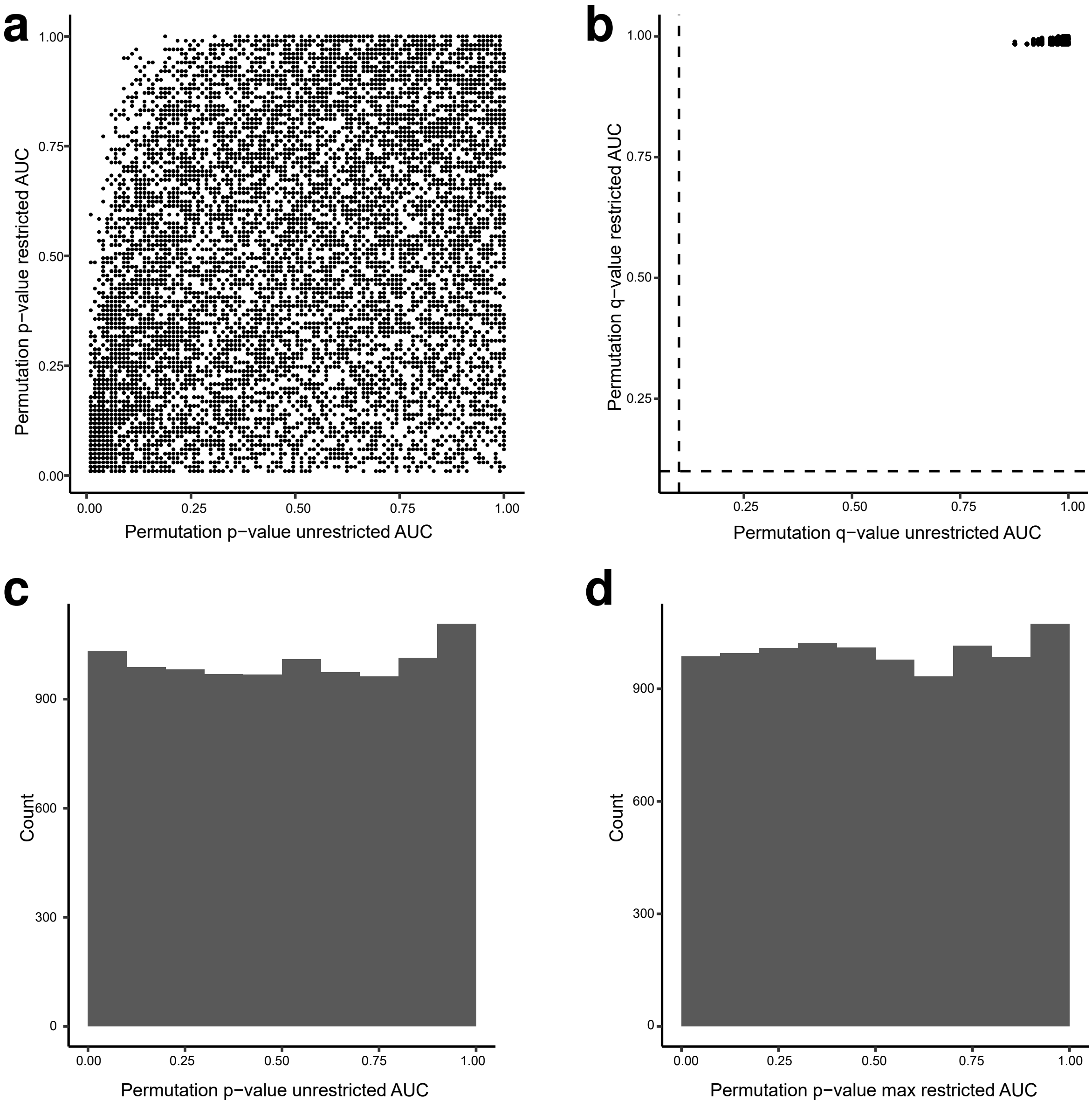

Supplementary Figure 7 *Restriction applied to randomly labelled data*

We used data from our training set of 110 patients with advanced melanoma. We selected the proportion of $CD4^{+}T_{\mathrm{EM}}$ cells as the biomarker of interest. We then randomly permuted the class labels of the samples and applied restriction to the permuted data. We repeated this procedure 10000 times to generate restricted and unrestricted permutation p-values. Correction for multiple testing with the false discovery rate led to a q-value of 1 for all biomarkers.

(a) Permutation p-values with (y-axis) and without (x-axis) restriction. Small unrestricted p-values tend to have smaller restricted p-values. This pattern arises from the fact that our method will result in no restriction being applied to the dataset if that yields better performance. Consequently, small unrestricted p-values also exhibit small restricted p-values. **(b)** Permutation q-values with (y-axis) and without (x-axis) restriction after p-value correction for multiple testing with the Benjamini-Hochberg procedure. Most of the q-values are 1, which is expected since the null hypothesis is true. **(c)** Permutation p-value distribution without restriction. We observe that the distribution is uniform as expected. There are 1033 biomarkers with an uncorrected permutation p-value below 0.1. **(d)** Permutation p-value distribution with restriction. We observe that the distribution is also uniform as expected. There are 987 biomarkers with an uncorrected permutation p-value below 0.1.

Supplementary Figure 8

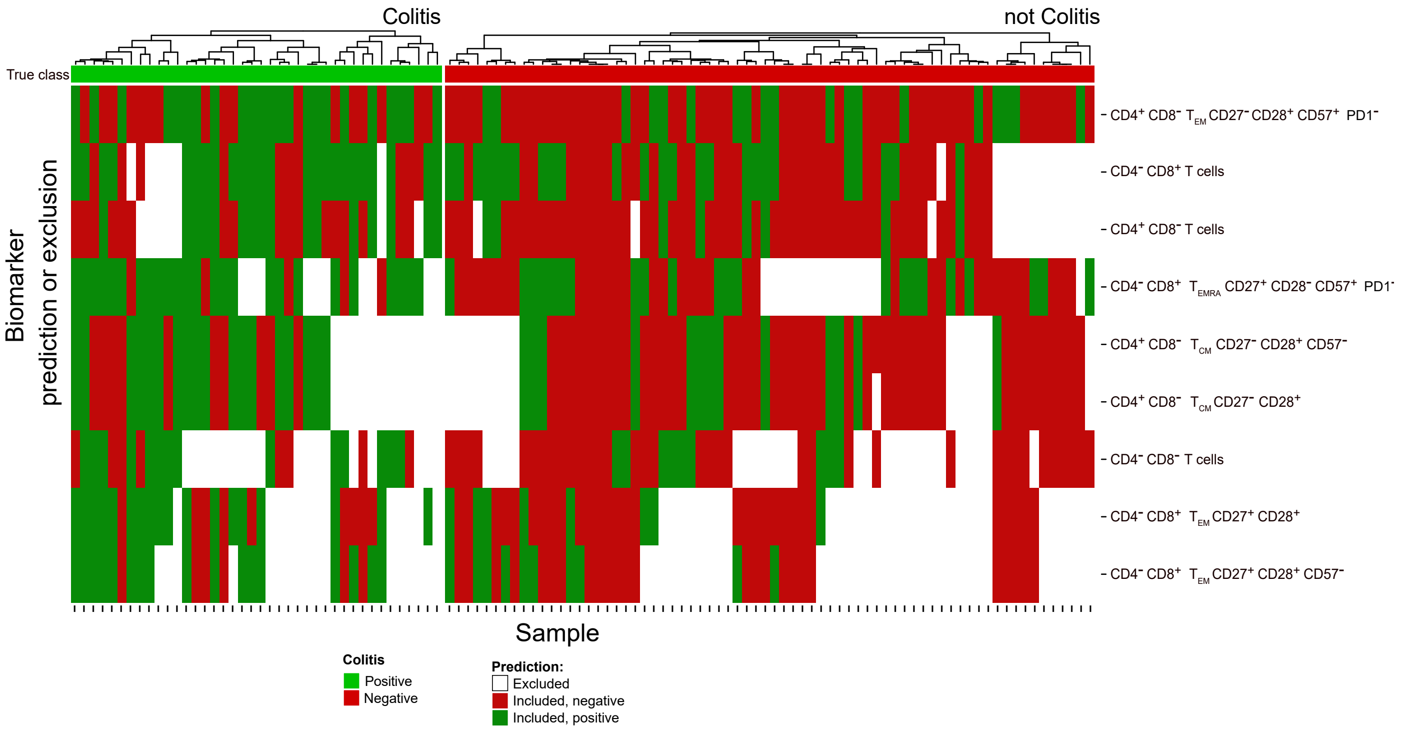

Supplementary Figure 8 *Significant biomarkers according to classical and restricted analysis predicting colitis.*

The heatmap shows the significant biomarkers for colitis based on permutation p-values for unrestricted AUC and our restriction method labelled on the right. No biomarker remained significant after correction for multiple testing. The biomarkers were cell populations from the DURAClone IM T Cell Subsets Tube with our gating strategy described in Figure 4a and Supplementary Figure 4. Each further column reflects one sample, each row a biomarker. The samples are grouped according to their true positive (green) or negative (red) class shown in the very first row. The main matrix consists of three values: 1) Excluded according to the restriction (white/empty), 2) included and predicted positive (dark green) and 3) included and predicted negative (dark red).

Supplementary Figure 9

a

- Proteomic data from Harel et al. 2019.
- FFPE samples from 74 patients treated with anti-PD1 immunotherapy.
- 4620 measured proteins.
- Objective: To find differentially expressed proteins between responders and non-responders defined by RECIST v1.0 guidelines.
- Restriction finds 2 otherwise missed significant proteins after correction for multiple testing at a q-value significance level of 0.1.

b

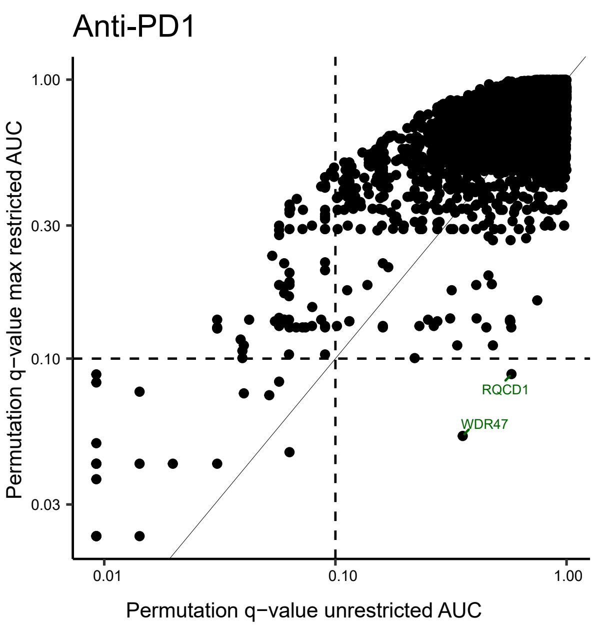

c

Biomarker	Restriction						Performance						
	Restricted?	AUC	P.value	Q.value	Inform.Range		Pred[>Cutoff]	Cutoff	NPV	PPV	TPR	TNR	ACC
					min	max							
WDR47	global	.655	.04574	.35546	-∞	∞	responder	-1.267	.787	.857	.545	.949	.803
WDR47	restricted	.905	.00016	.05280	-∞	-.401	responder	-1.267	.926	.857	.857	.926	.902
RQCD1	global	.602	.17132	.57772	-∞	∞	nonresponder	1.007	.731	.846	.440	.95	.754
RQCD1	restricted	.919	.00040	.08800	.62	∞	nonresponder	1.007	.900	.846	.846	.90	.879

d

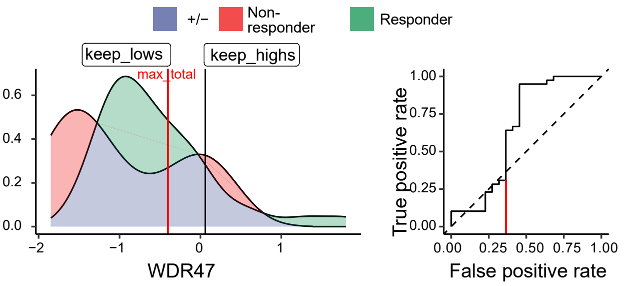

e

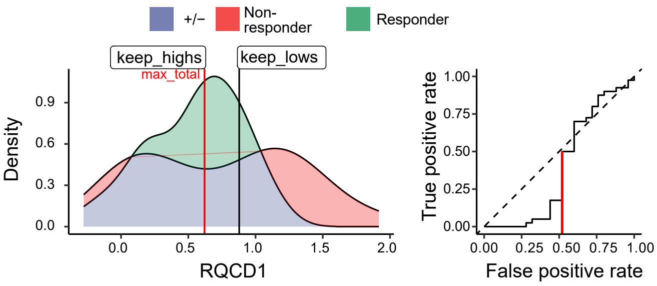

Supplementary Figure 9 *Restriction applied to proteomics - 1*

Supplementary Figure 9 Harel et al. (2019) investigated proteomic markers of response to anti-PD1 immunotherapy in patients with advanced melanoma. Using high-resolution mass spectrometry, they quantified 4620 proteins in formalin-fixed paraffin-embedded (FFPE) tissue samples from 74 patients treated with anti-PD1 immunotherapy. Patients are split into 40 responders, 27 non-responders, and 7 stable diseases according to RECIST v1.0 guidelines. We used the data available under Table S1 of the publication, sheets S1C and S1D. The authors were interested in significantly expressed proteins that discriminated between responders and non-responders: see Figure 3C of their publication. **(a)** Summary. **(b)** Permutation p-values for the restricted (y-axis) and unrestricted (x-axis) AUCs within the anti-PD1 cohort. **(c)** Table of significant biomarkers after restriction and correction for multiple testing (Benjamini-Hochberg). "Global" describes values when no restriction is applied. "Informative range" is the range of values where a meaningful classification is possible, for "global" within all observed values. Pred[>Cutoff] is the predicted class of a sample value within the informative range and above the discriminatory cutoff. Negative predictive value, positive predictive value, true positive rate (sensitivity), true negative rate (specificity), and accuracy are calculated within the informative range. **(d)** Densities, ROC curves and restriction value for WDR47. **(e)** Densities, ROC curves and restriction value for RQCD1.

Supplementary Figure 10

a

- Proteomic data from Harel et al. 2019.
- FFPE samples from 42 patients treated with TIL-based adoptive cell transfer.
- 4588 measured proteins.
- Objective: To find differentially expressed proteins between responders and non-responders defined by RECIST v1.0 guidelines.
- Restriction finds 2 otherwise missed significant proteins after correction for multiple testing at a q-value significance level of 0.1.

b

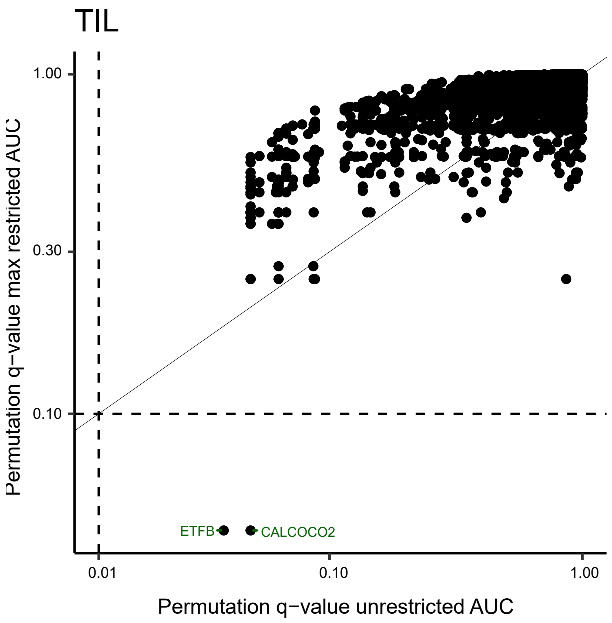

c

**Biomarker**	**Restriction**						**Performance**						
	Restricted?	AUC	P.value	Q.value	Inform.Range		Pred[>Cutoff]	Cutoff	NPV	PPV	TPR	TNR	ACC
					min	max							
ETFB	global	.862	4e-05	.18136	-∞	∞	responder	.832	.826	.895	.810	.905	.857
ETFB	restricted	.936	2e-05	.04534	.157	∞	responder	.832	.826	1.000	.789	1.000	.895
CALCOCO2	global	.851	3e-04	.20609	-∞	∞	responder	-.769	.824	.933	.824	.933	.875
CALCOCO2	restricted	.957	2e-05	.04534	-∞	.634	responder	-.769	.929	.933	.933	.929	.931

d

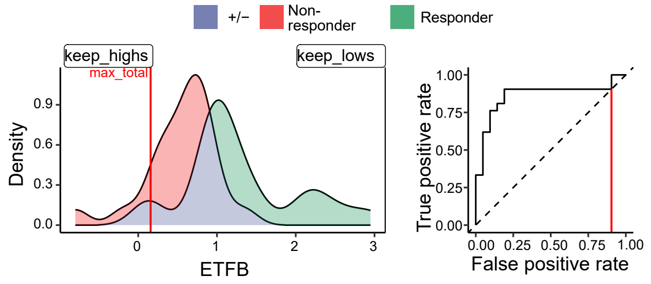

e

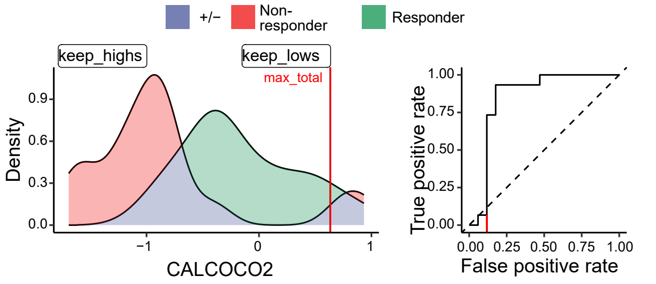

Supplementary Figure 10 *Restriction applied to proteomics - 2*

Supplementary Figure 10

Harel et al. (2019) investigated proteomic markers of response to TIL-based adoptive cell transfer in patients with advanced melanoma. Using high-resolution mass spectrometry, they quantified 4588 proteins in formalin-fixed paraffin-embedded (FFPE) tissue samples from 42 patients treated with anti-PD1 immunotherapy. Patients are split into 21 responders and 21 non-responders according to RECIST v1.0 guidelines. We used the data available under Table S1 of the publication, sheets S1C and S1D. The authors were interested in significantly expressed proteins that discriminated between responders and non-responders: see Figure 3C of their publication.

(a) Summary. **(b)** Permutation p-values for the restricted (y-axis) and unrestricted (x-axis) AUCs within the anti-PD1 cohort. **(c)** Table of significant biomarkers after restriction and correction for multiple testing (Benjamini-Hochberg). "Global" describes values when no restriction is applied. "Informative range" is the range of values where a meaningful classification is possible, for "global" within all observed values. Pred[>Cutoff] is the predicted class of a sample value within the informative range and above the discriminatory cutoff. Negative predictive value, positive predictive value, true positive rate (sensitivity), true negative rate (specificity), and accuracy are calculated within the informative range. **(d)** Densities, ROC curves, and restriction value for ETFB. **(e)** Densities, ROC curves and restriction value for CALCOCO2.

Supplementary Figure 11

a

- Microbiome data from Lee et al. 2022.
- A merged dataset of 312 faecal samples was analyzed, which comprised 165 new samples from 5 separate cohorts, plus 147 samples from four publicly available datasets.
- Across all samples, 668 species in 228 genera, 82 families, 42 orders, 26 classes and 14 phyla were identified. Here, we used all 1060 taxonomic identifiers as features for our reanalysis.
- Here, we used progression free survival (PFS12) as clinical endpoint for our reanalysis.
- Restriction finds 1 otherwise missed species and its parent genus after correction for multiple testing at a q-value significance level of 0.1.

b

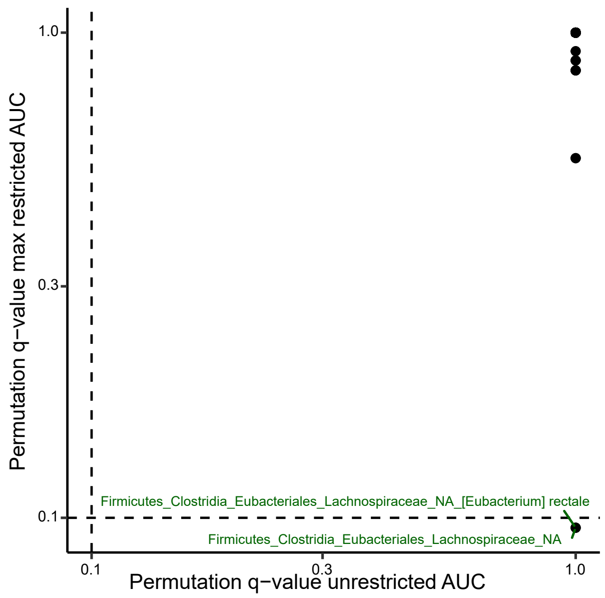

c

Biomarker	Restriction						Performance						
	Restricted?	AUC	P.value	Q.value	Inform.Range min	Inform.Range max	Pred[>Cutoff]	Cutoff	NPV	PPV	TPR	TNR	ACC
[Eubacterium] rectale*	global	.619	.00310	1.00000	-∞	∞	progression	-.026	.588	.725	.472	.808	.634
[Eubacterium] rectale*	restricted	.711	.00018	.09540	-∞	.762	progression	-.026	.655	.725	.625	.750	.686
Lachnospiraceae uncl.**	global	.620	.00294	1.00000	-∞	∞	progression	-.030	.588	.725	.472	.808	.634
Lachnospiraceae uncl.**	restricted	.712	.00018	.09540	-∞	.759	progression	-.030	.655	.725	.625	.750	.686

* Phylum:Firmicutes Class:Clostridia Order:Eubacteriales Family:Lachnospiraceae Genus:unclassified Species:[Eubacterium] rectale. Now known as species Agathobacter rectalis, NCBI:txid39491.

** Phylum:Firmicutes Class:Clostridia Order:Eubacteriales Family:Lachnospiraceae Genus:unclassified. Now known as genus Agathobacter, NCBI:txid1766253.

d

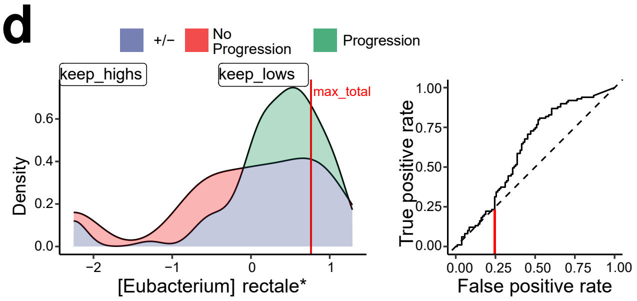

e

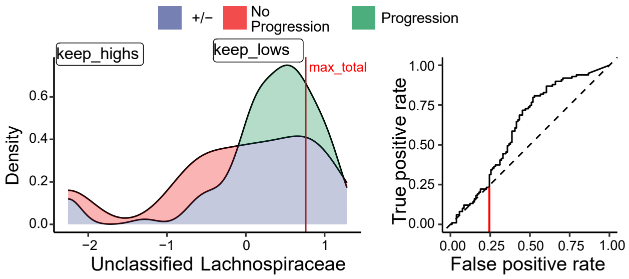

Supplementary Figure 11 *Restriction applied to microbiomics*

Supplementary Figure 11 Lee et al. (2022) describe the microbiome of faecal samples from 165 patients across five separate cohorts and an additional 147 samples from four publicly available datasets. Shotgun metagenomic sequencing was applied to capture information about 668 bacterial species. Progression free survival was obtained from the clinical data and was available for 205 of 312 samples. It was defined as the time from the first dose of an ICI to the first event; disease progression or death from any cause. Patients were labelled as "progression" if they had a progression event within 12 months of treatment initiation. **(a)** Summary. **(b)** Permutation p-values for the restricted (y-axis) and unrestricted (x-axis) AUCs within 205 labelled samples. **(c)** Table of significant biomarkers after restriction and correction for multiple testing (Benjamini-Hochberg). "Global" describes values when no restriction is applied. "Informative range" is the range of values where a meaningful classification is possible, for "global" within all observed values. Pred[>Cutoff] is the predicted class of a sample value within the informative range and above the discriminatory cutoff. Negative predictive value, positive predictive value, true positive rate (sensitivity), true negative rate (specificity) and accuracy are calculated within the informative range. **(d)** Densities, ROC curves and restriction value for species Agathobacter rectalis. **(e)** Densities, ROC curves and restriction value for Lachnospiraceae incertae sedis.

Supplementary Figure 12

a

- Mass cytometry data from Lozano et al. 2022.
- 18 pre-treatment peripheral blood samples from patients with metastatic melanoma treated with ICI-based immunotherapy.
- CyTOF data were analyzed with FlowSOM and 20 resulting subpopulations of major mononuclear lineages were annotated.
- Outcome was defined as severe immune related adverse event after treatment initiation.
- Restriction finds 2 otherwise missed subpopulations after correction for multiple testing at a q-value significance level of 0.1.

b

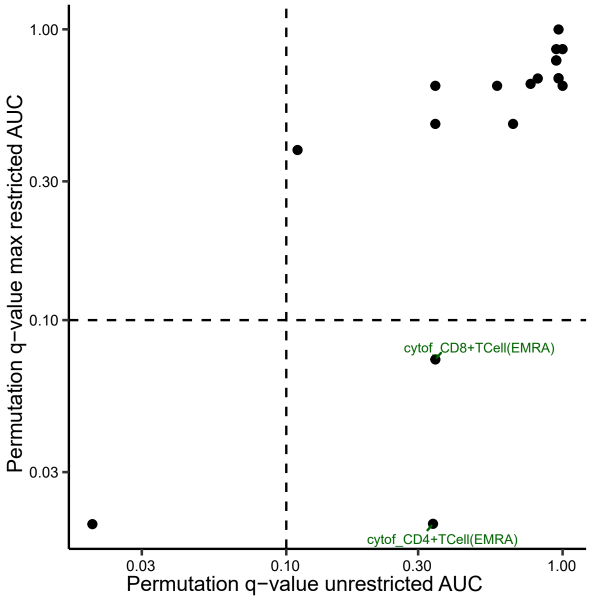

c

Biomarker	Restriction						Performance						
	Restricted?	AUC	P.value	Q.value	Inform.Range		Pred[>Cutoff]	Cutoff	NPV	PPV	TPR	TNR	ACC
					min	max							
CD4$^+$ T$_{EMRA}$	global	.763	.05094	.33962	-∞	∞	1	.012	.667	1	.60	1	.778
CD4$^+$ T$_{EMRA}$	restricted	.953	.00199	.01993	-∞	.042	1	.012	.800	1	.75	1	.875
CD8$^+$ T$_{EMRA}$	global	.737	.10389	.34630	-∞	∞	0	.025	.727	1	.7	1	.833
CD8$^+$ T$_{EMRA}$	restricted	1.000	.01098	.07322	.01	∞	0	.025	1.000	1	1.0	1	1.000

d

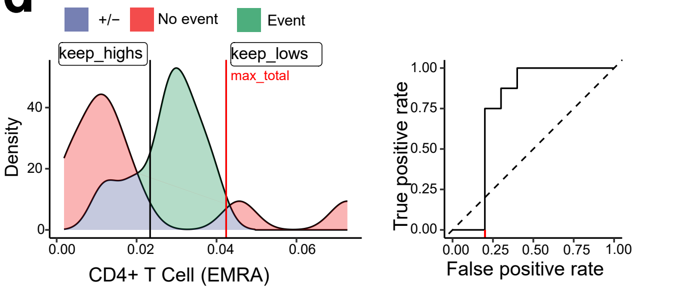

e

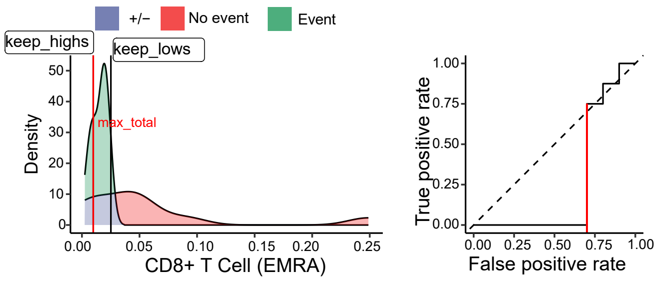

Supplementary Figure 12 *Restriction applied to mass cytometry*

Supplementary Figure 12 Lozano et al. (2022) attempted to find markers of severe immune-related adverse events (irAEs) after ICI therapy in patients with metastatic melanoma. Pretreatment blood from 18 samples with 8 severe and 10 non-severe irAEs were measured with CyTOF. Samples were labelled according to CTCAE v5.0 where grade ≥ 3 was considered severe irAE. The outcome was defined as severe irAE after treatment initiation. Patients were treated with anti-PD1 (n=4) or anti-PD1 + anti-CTLA4 (n=14). CyTOF data were analyzed with FlowSOM resulting in 20 subpopulations of major mononuclear lineages. **(a)** Summary. **(b)** Permutation p-values for the restricted (y-axis) and unrestricted (x-axis) AUCs within 18 samples. **(c)** Table of significant biomarkers after restriction and correction for multiple testing (Benjamini-Hochberg). "Global" describes values when no restriction is applied. "Informative range" is the range of values where a meaningful classification is possible, for "global" within all observed values. Pred[>Cutoff] is the predicted class of a sample value within the informative range and above the discriminatory cutoff. Negative predictive value, positive predictive value, true positive rate (sensitivity), true negative rate (specificity) and accuracy are calculated within the informative range. **(d)** Densities, ROC curves and restriction value for the proportion of $CD4^+$ T_{EMRA} cells among total evaluable PBMCs. **(e)** Densities, ROC curves and restriction value for the proportion of $CD8^+$ T_{EMRA} cells among total evaluable PBMCs.

Supplementary Figure 13

a

- Transcriptomic data from Zhang et al. 2022.
- 921 transcriptomic samples from 10 ICI RNA-seq cohorts were split into 618 training, 154 validation (studies 1-5) and 149 test (studies 6-10) samples.
- A total of 15,896 genes were quantified.
- Outcomes were defined as objective response rate (ORR) according to RECIST v1.1 or irRECIST criteria.
- Here we report univariate restriction applied to 15896 measured genes. Restriction finds 19 otherwise missed genes after correction for multiple testing at a q-value significance level of 0.1. We show the performances of 4 selected genes.

b

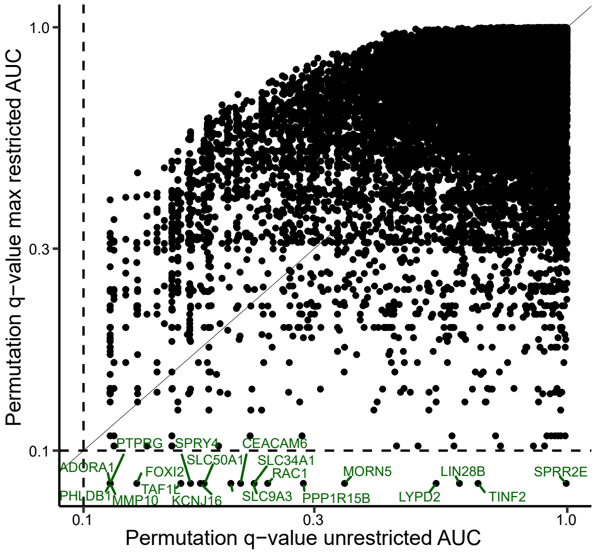

c

**Biomarker**	**Restriction**							**Performance**						
	Restricted?	Data	AUC	P.value	Q.value	Inform.Range		Pred[>Cutoff]	Cutoff	NPV	PPV	TPR	TNR	ACC
						min	max							
LYPD2	global	train	.539	.13339	.53570	-∞	∞	NR	-2.651	.810	.310	.745	.395	.489
		val	.484							.791	.310	.659	.469	.519
		test	.512							.684	.413	.679	.419	.517
	restricted	train	.642	.00010	.08365	-6.204	∞	NR	-2.651	.810	.393	.625	.624	.624
		val	.685							.791	.537	.611	.736	.694
		test	.606							.684	.491	.600	.582	.589
CEACAM6	global	train	.571	.00800	.21122	-∞	∞	NR	-.257	.810	.336	.661	.525	.561
		val	.500							.767	.296	.585	.496	.519
		test	.458							.627	.378	.554	.452	.490
	restricted	train	.631	.00010	.08365	-2.752	∞	NR	-.257	.810	.393	.606	.642	.632
		val	.644							.767	.431	.564	.659	.629
		test	.486							.627	.408	.537	.500	.514
RAC1	global	train	.568	.01140	.24031	-∞	∞	R	4.281	.807	.330	.667	.508	.550
		val	.534							.792	.317	.634	.504	.539
		test	.557							.663	.420	.518	.570	.550
	restricted	train	.664	.00010	.08365	-∞	4.613	R	4.281	.807	.434	.534	.737	.681
		val	.593							.792	.394	.464	.740	.667
		test	.547							.662	.409	.250	.803	.608
SPRR2E	global	train	.501	.98180	.99507	-∞	∞	R	-7.469	.789	.302	.697	.413	.489
		val	.659							.915	.379	.878	.478	.584
		test	.514							.600	.375	.964	.032	.383
	restricted	train	.646	.00010	.08365	-∞	-5.634	R	-7.469	.789	.446	.537	.722	.668
		val	.876							.915	.583	.808	.783	.789
		test	.566							.600	.398	.949	.051	.408

Supplementary Figure 13 *Restriction applied to transcriptomics*

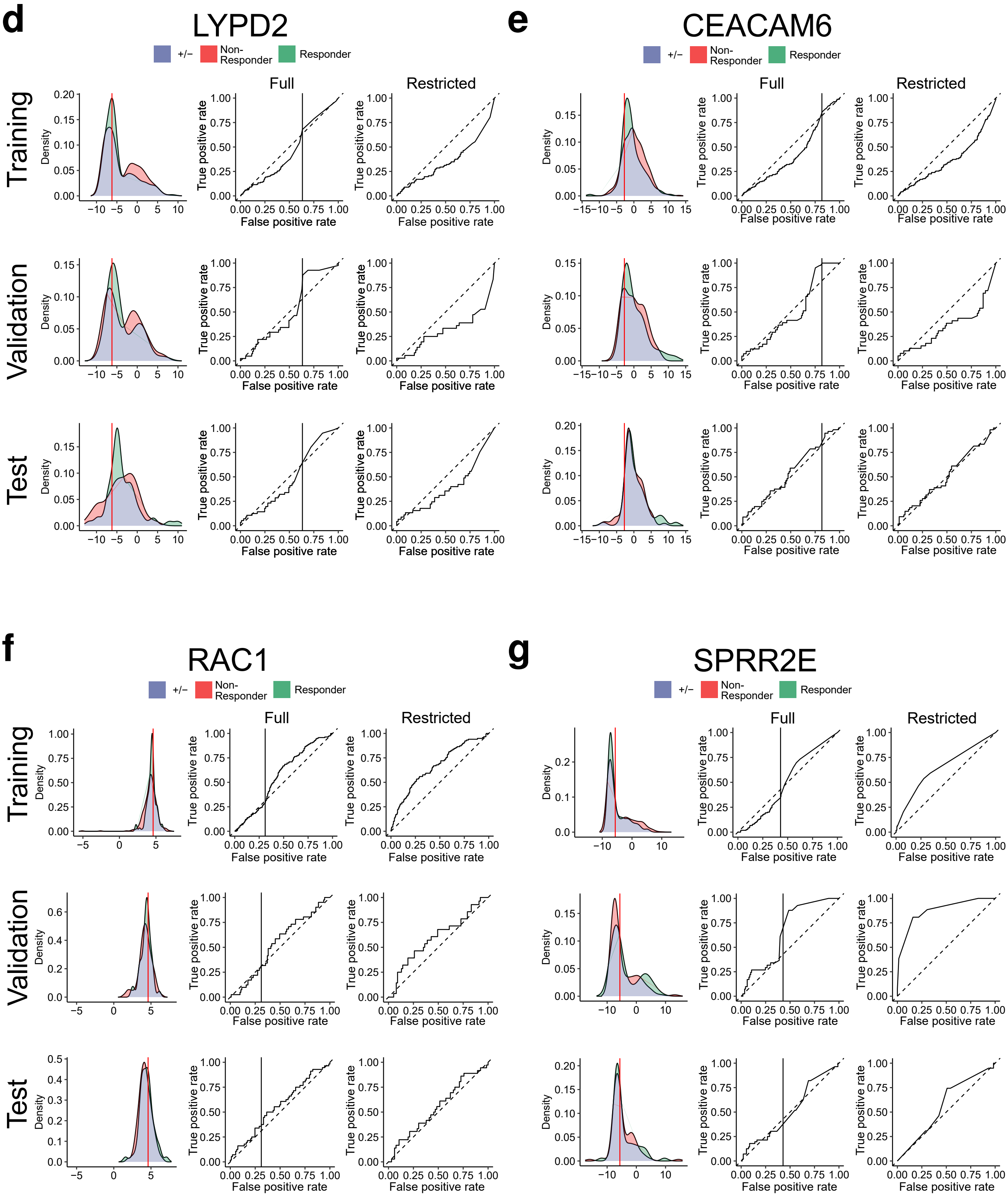

Supplementary Figure 13

Supplementary Figure 13 Zhang et al. (2022) gathered data from 10 studies about patients treated with immune checkpoint inhibitors. 15896 genes were quantified in 618 training, 154 validation and 149 test bulk RNA-seq samples. Objective response rate was defined according to RECIST v1.1 or irRECIST criteria in one study. We established univariate restriction on the training samples and evaluated it on training, validation and test sets. The training and validation samples were from five studies and randomly split into 80% training and 20% validation samples. The test samples were from five other studies. All bulk samples originated from five cancer types and were treated with one of four ICI treatments.

(a) Summary. **(b)** Permutation p-values for the restricted (y-axis) and unrestricted (x-axis) AUCs within 618 training samples. **(c)** Table of significant biomarkers after restriction and correction for multiple testing (Benjamini-Hochberg). "Global" describes values when no restriction is applied. "Informative range" is the range of values where a meaningful classification is possible, for "global" within all observed values. Pred[>Cutoff] is the predicted class of a sample value within the informative range and above the discriminatory cutoff. Negative predictive value, positive predictive value, true positive rate (sensitivity), true negative rate (specificity) and accuracy are calculated within the informative range. **(d-g)** Density and ROC curve with restriction value as well as restricted ROC curve for training (618), validation (154) and test (149) samples. LYPD2, CEACAM6, RAC1 and SPRR2E are shown as examples.

Supplementary Figure 14

a

- Transcriptomic data from Zhang et al. 2022.
- 921 transcriptomic samples from 10 ICI RNA-seq cohorts were split into 618 training, 154 validation (studies 1-5) and 149 test (studies 6-10) samples.
- A total of 15,896 genes were quantified, but following the analysis in Figure 4 of Zhang et al. 2022, only 369 genes from the initially established "Stem.Sig" gene signature were used.
- Multiple machine learning models were trained and validated on the respective data splits to predict the objective response rate (ORR) according to RECIST v1.1 or irRECIST criteria.
- Here we report multivariate random forest model performances with and without preprocessing the genes by restriction. We find that the validation and test set performances improve with restriction.

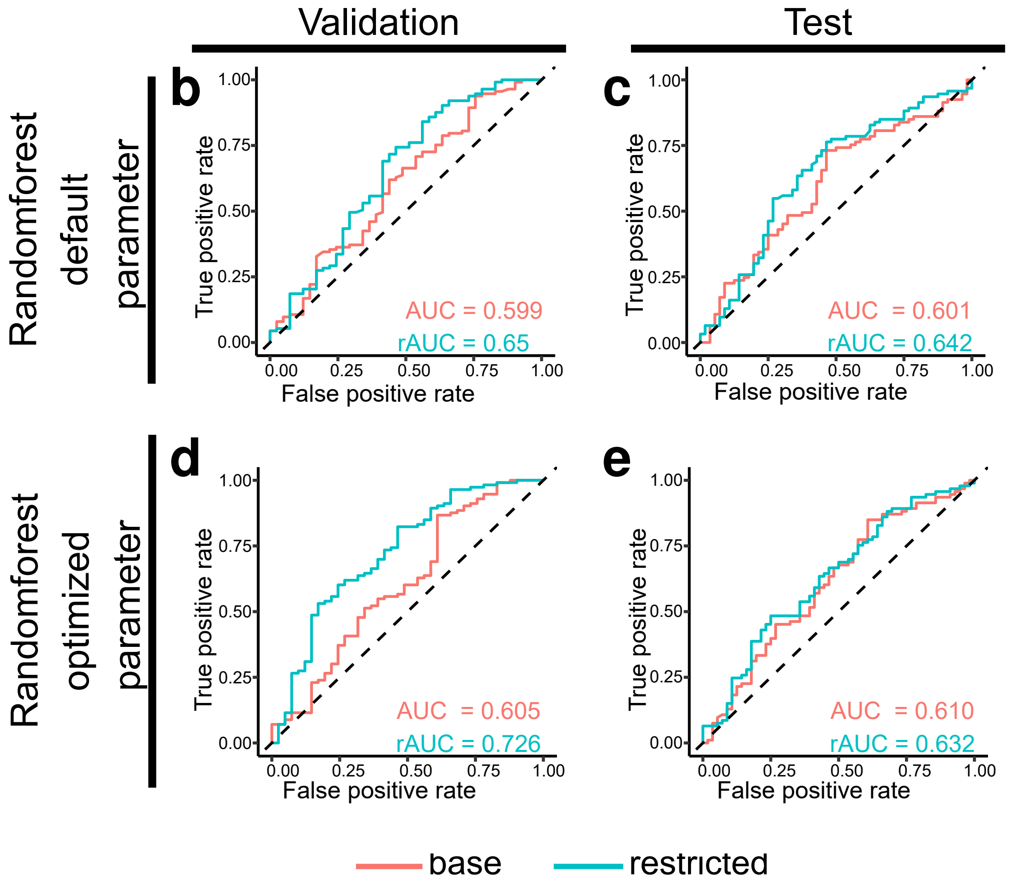

Supplementary Figure 14 *Multivariate restriction applied to transcriptomics*

Supplementary Figure 14 Zhang et al. (2022) built multiple machine learning models to predict objective response rates in immune checkpoint inhibitor-treated patients. They gathered single-cell data from 345 patients in 17 cancer types to find the "Stem.Sig" gene signature, a list of 454 differentially expressed genes between non-/malignant cells identified by CytoTRACE. In accordance with the article, we built multivariate random forests on the subset of 369 genes, identified in the 921 bulk RNA-seq measurements. Zhang et al. (2022) partitioned the dataset into 618 bulk RNA-seq training samples, 154 validation and 149 test samples. The training and validation samples were from five studies and randomly split into 80% training and 20% validation samples. The test samples were from five other studies. All bulk samples originated from five cancer types and were treated with one of four ICI treatments. The clinical outcome objective response rate (ORR) was defined according to RECIST v1.1 or irRECIST criteria in one study. Patients were classified as responders (complete response or partial response) or non-responders (stable disease or progressive disease).

(a) Summary. **(b-e)** ROC curves of restricted (blue) and base (red) random forest models on validation (left) and test (right) samples with (bottom) and without (top) optimization of random forest hyperparameters. Default parameters were set as 1000 trees, a maximum depth of 20, a minimum number of leaf observations of 1, 20 bins, 21 random candidate biomarkers per split and a sample rate of 0.632. Parameter optimization was performed with a maximum of 500 models, random parameter selection of 50-1000 trees, 3-10 random candidate biomarkers per split, a depth of 5-30, 1-3 minimum number of leaf observations of 1-3, 20-50 bins and a sample rate of 0.55-0.85. The imputed value for samples outside the informative range was defined as -20, being outside the range of all values across all samples.

Supplementary Figure 15

a

- Transcriptomic data from Zhang et al. 2022.
- 921 transcriptomic samples from 10 ICI RNA-seq cohorts were randomly split into 70% training and 30% validation samples.
- A total of 15,896 genes were quantified, but following the analysis in Figure 4 of Zhang et al. 2022, only 369 genes from the initially established “Stem.Sig” gene signature were used.
- Here we report multivariate random forest model performances with and without preprocessing the genes by restriction for 750 random splits into training (70%) and test (30%) set. We find that the test set performance improves with restriction preprocessing.

b

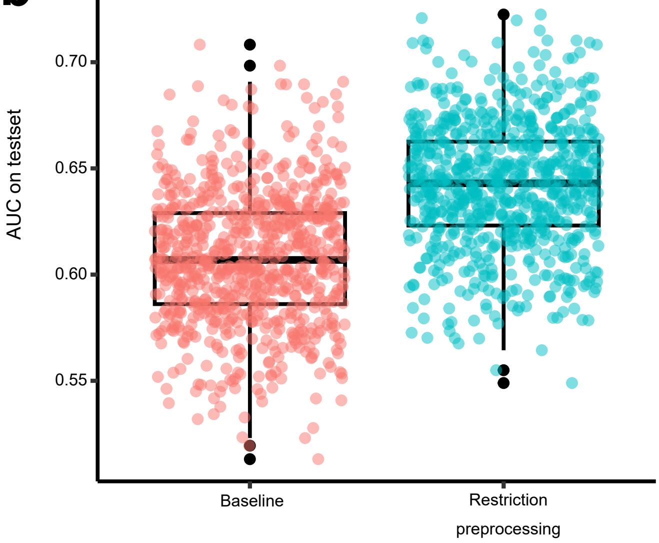

c

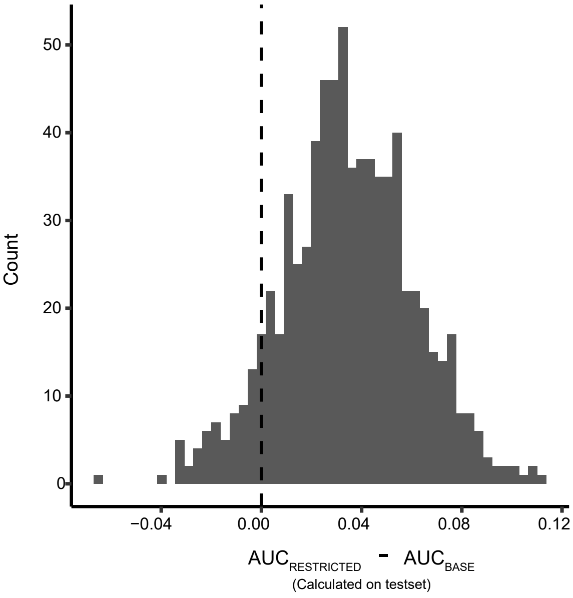

Supplementary Figure 15 *Multivariate restriction applied to transcriptomics with random train/test splits*
Zhang et al. (2022) built multiple machine learning models to predict objective response rates in immune checkpoint inhibitor-treated patients. We used 369 genes from their "Stem.Sig" gene signature as potential biomarkers. We repeatedly (750 times) split the 921 measured bulk RNA-seq samples into training (70%) and test (30%) sets. For each split, the baseline model built a random forest model on the training set ("Baseline") and applied it to the test data. "Restriction preprocessing" first established the restriction values on the training set, applied them to restrict the training set and built a random forest model on this restricted data. Then the restriction values were applied and the model was evaluated on the test set. All bulk samples originated from five cancer types and were treated with one of four ICI treatments. The clinical outcome objective response rate (ORR) was defined according to RECIST v1.1 or irRECIST criteria in one study. Patients were classified as responders (complete response or partial response) or non-responders (stable disease or progressive disease). We used default parameters for the random forest: 1000 trees, a maximum depth of 20, a minimum number of leaf observations of 1, 20 bins, 21 random candidate biomarkers per split and a sample rate of 0.632.
(a) Summary. **(b)** AUCs as points and summarized by boxplots for baseline (red) and restriction preprocessing (blue) on the test set for 750 random splits into training and test set. **(c)** Histogram of the differences between AUCs of restriction preprocessing and baseline on the test set for 750 random splits into training and test set.

Supplementary Table 1

	Training Set	Validation Set
Total number of cases	110	30
Enrollment	10/2016 - 06/2021	06/2021 - 01/2023
Female	37 (33.6 %)	12 (40.0 %)
Male	73 (66.4 %)	18 (60.0 %)
Age (years)	62 (22-84)	64 (23-84)
BMI	26.6 (15.4-54.6)	28.3 (15.0-44.6)
Stage III	8 (7.3 %)	1 (3.3 %)
Stage IV	102 (92.7 %)	29 (96.7 %)
Liver metastases present	30 (27.3 %)	9 (30.0 %)
Pretreatment		
None	3 (2.7 %)	1 (3.3 %)
Surgical excision	102 (92.7%)	29 (96.7 %)
Radiosurgery	3 (2.7 %)	0 (0.0 %)
Radiation	42 (38.2 %)	6 (20.0 %)
Monotherapy	17 (15.5 %)	5 (16.7 %)
IFNa therapy	9 (8.2 %)	2 (6.7 %)
Braf/Mek inhibitor therapy	21 (19.1 %)	4 (13.3 %)
T-VEC therapy	7 (6.4 %)	0 (0.0 %)
Chemotherapy	6 (5.5 %)	3 (10.0 %)
Rounds of Ipi/Nivo		
1 round	13 (11.8 %)	5 (16.7 %)
2 rounds	24 (21.8 %)	2 (6.7 %)
3 rounds	20 (18.2 %)	9 (30.0 %)
4 rounds	53 (48.2 %)	14 (46.7 %)
Complications		
No complication	35 (31.8 %)	6 (20.0 %)
Hepatitis	48 (43.6 %)	16 (53.3 %)
Colitis	40 (36.4 %)	12 (40.0 %)
Hepatitis and Colitis	13 (11.8 %)	4 (13.3 %)

Supplementary Table 1 *Patient cohort characteristics of training and validation set.* EDTA blood samples of 140 stage III/IV melanoma patients were collected from OCT-2016 until JAN-2023. Data from 110 training set patients have been published previously (Glehr, 2022). For age and BMI, median values were calculated. Minimum and maximum values are given in brackets. Patient cohort characteristics were obtained at start of Ipi/Nivo therapy. Male and female sex were self-reported.

Supplementary Note 3: ROC definition and probabilistic interpretation

3.1 Definition of the ROC

Let a threshold $c \in \mathbb{R}$, a continuous biomarker $Y \in \mathbb{R}$ and a grouping of samples into diseased (positive, $D = 1$) and non-diseased (negative, $D = 0$). A sample can be classified into diseased if $Y \geq c$ and into non-diseased if $Y < c$. The true positive rate (TPR) and false positive rate (FPR) at threshold c are defined as

$$\mathrm{TPR}(c) = \mathrm{P}[Y \geq c \mid D = 1], \tag{1}$$
$$\mathrm{FPR}(c) = \mathrm{P}[Y \geq c \mid D = 0]. \tag{2}$$

The ROC curve connects the TPR and FPR for all possible thresholds. We can write the set of points of the ROC curve as

$$\mathrm{ROC}_{set} = \left\{ \begin{pmatrix} \mathrm{FPR}(c) \\ \mathrm{TPR}(c) \end{pmatrix}, c \in (-\infty, \infty) \right\}. \tag{3}$$

With Equations (1) and (2), as the threshold c increases, both $\mathrm{FPR}(c)$ and $\mathrm{TPR}(c)$ decrease, see Supplementary Figure 16:

$$\lim_{c\to\infty} \mathrm{TPR}(c) = 0, \qquad \lim_{c\to-\infty} \mathrm{TPR}(c) = 1, \qquad \lim_{c\to\infty} \mathrm{FPR}(c) = 0, \qquad \lim_{c\to-\infty} \mathrm{FPR}(c) = 1, \tag{4}$$

therefore

$$\lim_{c\to\infty} \mathrm{ROC}_{set}(c) = \left\{ \begin{pmatrix} 0 \\ 0 \end{pmatrix} \right\}, \qquad \lim_{c\to-\infty} \mathrm{ROC}_{set}(c) = \left\{ \begin{pmatrix} 1 \\ 1 \end{pmatrix} \right\}. \tag{5}$$

The ROC curve is a monotonically increasing function. After substitution in Equation (3)

$$t := \mathrm{FPR}(c) \iff \mathrm{FPR}^{-1}(t) = c, \tag{6}$$

where c is the threshold corresponding to the false positive rate t such that $P[Y \geq c|D = 0] = t$. Here we assume that FPR is strictly monotonic such that it is invertible. Then

$$\mathrm{ROC}_{set} = \left\{ \begin{pmatrix} \mathrm{FPR}\left(\mathrm{FPR}^{-1}(t)\right) \\ \mathrm{TPR}(\mathrm{FPR}^{-1}(t)) \end{pmatrix}, t \in (0, 1) \right\}, \tag{7}$$
$$= \left\{ \begin{pmatrix} t \\ \mathrm{ROC}(t) \end{pmatrix}, t \in (0, 1) \right\}, \tag{8}$$

with the definition

$$\mathrm{ROC}(t) := \mathrm{TPR}(\mathrm{FPR}^{-1}(t)). \tag{9}$$

We simplify notation:

$$\mathrm{P}\left[Y_D \geq y\right] := \mathrm{P}\left[Y \geq y \mid D = 1\right], \tag{10}$$
$$\mathrm{P}\left[Y_{\bar{D}} \geq y\right] := \mathrm{P}\left[Y \geq y \mid D = 0\right]. \tag{11}$$

We describe the empirical survival functions from given diseased and non-diseased values

$$S_D(y) := \mathrm{P}\left[Y_D \geq y\right] = \int_y^\infty f_D(u)du \mathrel{\hat{=}} \mathrm{TPR}(y), \tag{12}$$
$$S_{\bar{D}}(y) := \mathrm{P}\left[Y_{\bar{D}} \geq y\right] = \int_y^\infty f_{\bar{D}}(u)du \mathrel{\hat{=}} \mathrm{FPR}(y), \tag{13}$$

where $f_D(y)$ and $f_{\overline{D}}(y)$ are the probability density functions of Y_D and $Y_{\overline{D}}$, respectively. Their derivatives are the negative probability density functions:

$$\frac{\mathrm{d}S_D(y)}{\mathrm{d}y} = -f_D(y), \qquad \frac{\mathrm{d}S_{\overline{D}}(y)}{\mathrm{d}y} = -f_{\overline{D}}(y). \tag{14}$$

In terms of the survival functions,

$$\mathrm{ROC}(t) = S_D\left(S_{\overline{D}}^{-1}(t)\right), t \in (0,1), \tag{15}$$

and following Equation (6),

$$P[Y_D \geq S_D^{-1}(t)] = t, \tag{16}$$

$$P[Y_{\overline{D}} \geq S_{\overline{D}}^{-1}(t)] = t. \tag{17}$$

We define

$$f_{D\overline{D}}(u, y) := f_D(u) \cdot f_{\overline{D}}(y), \tag{18}$$

as the joint probability density function of Y_D and $Y_{\overline{D}}$ for independent random variables Y_D and $Y_{\overline{D}}$.

We further describe the following probability in terms of the empirical survival functions:

$$P[a \leq Y_X \leq b] = \int_a^b f_X(y)dy \tag{19}$$

$$= \int_a^\infty f_X(y)dy - \int_b^\infty f_X(y)dy \tag{20}$$

$$= S_X(a) - S_X(b), \qquad X \in \{D, \overline{D}\}. \tag{21}$$

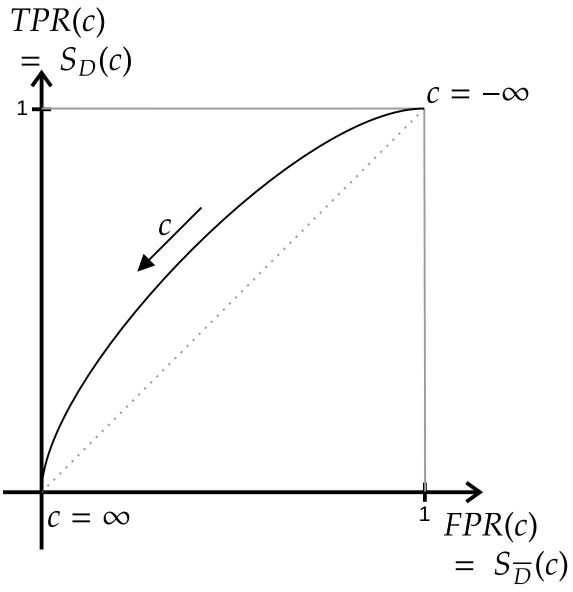

Supplementary Figure 16 *ROC curve with explicit threshold notation*

We show the ROC curve using notations used during the proof. The ROC curve shows the true positive rate $TPR(c)$ or empirical survival function for diseased $S_D(c)$ on the y-axis and the false positive rate $FPR(c)$ or empirical survival function for non-diseased $S_{\overline{D}}(c)$ on the x-axis for any possible threshold c. Samples with higher values than c are classified as diseased (or positive), and samples with smaller or equal values are classified as non-diseased (or negative). An infinitely high positive threshold predicts all samples as negative, resulting in a true positive rate and a false positive rate of 0. Conversely, an infinitely low negative threshold predicts all samples as positive, resulting in a true positive rate and false positive rate of 1.

3.2 The area under the ROC curve (AUC)

The area under the ROC curve is defined as

$$\mathrm{AUC} = \int_0^1 \mathrm{ROC}(t)\mathrm{d}t, \tag{22}$$

where a perfect ROC curve has an AUC of 1. "Perfect" corresponds to a perfectly discriminating biomarker where higher values correspond to the positive class. An uninformative biomarker has an AUC of 0.5 because $\mathrm{ROC}(t) = t$ for $t \in (0,1)$. A perfectly discriminating biomarker with higher values corresponding to the *negative* class has an AUC of 0.

From a probabilistic point of view, the AUC equals the probability that the biomarker value of a random positive sample is higher than that of a random negative sample (Bamber, 1975; Hanley and McNeil, 1982):

$$\mathrm{AUC} = \mathrm{P}\left[Y_D > Y_{\overline{D}}\right]. \tag{23}$$

Proof

$$\mathrm{AUC} = \int_0^1 \mathrm{ROC}(t)\mathrm{d}t \tag{24}$$

$\big\downarrow$ With Equation (15)

$$= \int_0^1 S_D\left(S_{\overline{D}}^{-1}(t)\right)\mathrm{d}t \tag{25}$$

$\big\downarrow$ Substitute $y := S_{\overline{D}}^{-1}(t) \iff t = S_{\overline{D}}(y)$
With Equation (5):

$$S_{\overline{D}}^{-1}(1) = -\infty$$
$$S_{\overline{D}}^{-1}(0) = \infty$$

$$= \int_{S_{\overline{D}}^{-1}(0)=\infty}^{S_{\overline{D}}^{-1}(1)=-\infty} S_D(y)S'_{\overline{D}}(y)\mathrm{d}y$$

$\big\downarrow$ With Equation (13) and Equation (14)

$$= \int_{\infty}^{-\infty} S_D(y)(-f_{\overline{D}}(y))\mathrm{d}y \tag{26}$$

$\big\downarrow$ With Equation (12)

$$= \int_{-\infty}^{\infty}\int_y^{\infty} f_D(u)\mathrm{d}u f_{\overline{D}}(y)\,\mathrm{d}y \tag{27}$$

$$= \int_{-\infty}^{\infty}\int_y^{\infty} f_D(u)f_{\overline{D}}(y)\mathrm{d}u\,\mathrm{d}y \tag{28}$$

$\big\downarrow$ With Equation (18)

$$= \int_{-\infty}^{\infty}\int_y^{\infty} f_{D\overline{D}}(u,y)\mathrm{d}u\,\mathrm{d}y \tag{29}$$

$$= \mathrm{P}\left[Y_D \geq Y_{\overline{D}}\right] \tag{30}$$

Supplementary Note 4: Restriction method

4.1 Partial area under the ROC curve (pAUC)

The partial AUC (pAUC) has been defined as the AUC up to a certain false positive rate t_0 and its probabilistic correspondence has been shown (Pepe 2004, Dodd 2001):

$$\mathrm{pAUC}(t_0) = \int_0^{t_0} \mathrm{ROC}(t)\mathrm{d}t = t_0 \cdot \mathrm{P}\left[Y_D > Y_{\bar{D}} | Y_{\bar{D}} > S_{\bar{D}}^{-1}(t_0)\right], \tag{31}$$

$$\mathrm{pAUC}(t_0) = \begin{cases} \int_0^{t_0} 1\mathrm{d}t = t_0 & \text{perfect test} \\ \int_0^{t_0} t\mathrm{d}t = \frac{t_0^2}{2} & \text{uninformative test.} \end{cases} \tag{32}$$

$$\mathrm{pAUC}(t_0) = \int_0^{t_0} \mathrm{ROC}(t)\mathrm{d}t \tag{33}$$

↓ With Equation (15)

$$= \int_0^{t_0} S_D\left(S_{\bar{D}}^{-1}(t)\right)\mathrm{d}t \tag{34}$$

↓ Substitute $y := S_{\bar{D}}^{-1}(t) \iff t = S_{\bar{D}}(y)$
With Equation (5):

$$S_{\bar{D}}^{-1}(0) = \infty$$

$$= \int_{S_{\bar{D}}^{-1}(0)=\infty}^{S_{\bar{D}}^{-1}(t_0)} S_D(y)S'_{\bar{D}}(y)\mathrm{d}y \tag{35}$$

↓ With Equation (13) and Equation (14)

$$= \int_{\infty}^{S_{\bar{D}}^{-1}(t_0)} S_D(y)(-f_{\bar{D}}(y))\mathrm{d}y$$

↓ With Equation (12)

$$= \int_{S_{\bar{D}}^{-1}(t_0)}^{\infty} \int_y^{\infty} f_D(u)\mathrm{d}u f_{\bar{D}}(y)\,\mathrm{d}y \tag{36}$$

$$= \int_{S_{\bar{D}}^{-1}(t_0)}^{\infty} \int_y^{\infty} f_D(u) f_{\bar{D}}(y)\mathrm{d}u\,\mathrm{d}y \tag{37}$$

↓ With Equation (18)

$$= \int_{S_{\bar{D}}^{-1}(t_0)}^{\infty} \int_y^{\infty} f_{D\bar{D}}(u,y)\mathrm{d}u\,\mathrm{d}y \tag{38}$$

$$= \mathrm{P}\left[Y_D \geq Y_{\bar{D}}, Y_{\bar{D}} \geq S_{\bar{D}}^{-1}(t_0)\right] \tag{39}$$

$$= \mathrm{P}\left[Y_{\bar{D}} \geq S_{\bar{D}}^{-1}(t_0)\right] \mathrm{P}\left[Y_D \geq Y_{\bar{D}} | Y_{\bar{D}} \geq S_{\bar{D}}^{-1}(t_0)\right] \tag{40}$$

↓ with Equation (16): $\mathrm{P}\left[Y_{\bar{D}} \geq S_{\bar{D}}^{-1}(t_0)\right] = t_0$

$$\mathrm{pAUC}(t_0) = t_0 \cdot \mathrm{P}\left[Y_D \geq Y_{\bar{D}} | Y_{\bar{D}} \geq S_{\bar{D}}^{-1}(t_0)\right] \tag{41}$$

4.2 Two-way partial area under the ROC curve

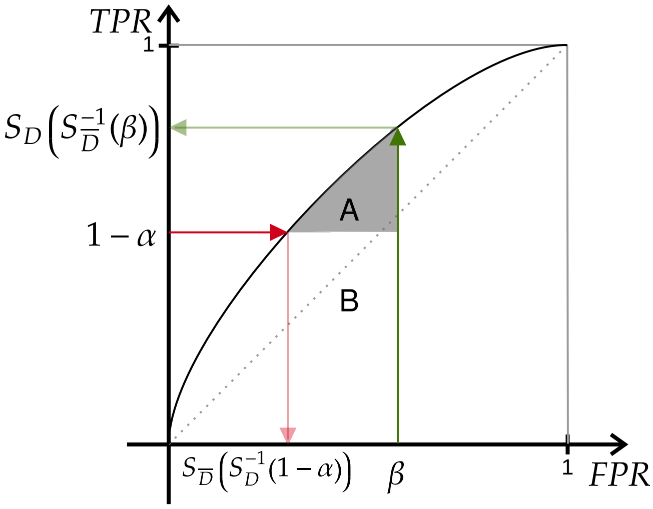

Supplementary Figure 17 *Two-way partial area under the ROC curve.*
The two-way partial area under the ROC curve is defined as the area under the ROC curve between a maximum false positive rate β and a minimum true positive rate $1-\alpha$. Alternatively, calculate the area under the curve between the corresponding false positive rates $\left[S_{\bar{D}}(S_D^{-1}(1-\alpha)), \beta\right]$ and subtract the area of the rectangle B.

The concept of the partial area under the curve (pAUC) has been expanded in recent years to include two-way partial AUCs (Yang 2017 and Yang 2021), which allow for the calculation of the area under the curve between an upper limit for the false positive rate and a lower limit for the true positive rate ($S_{\bar{D}}(t) \leq \beta$ and $S_D(t) \geq 1-\alpha$). This area, shown in Supplementary Figure 17 as shaded area A, can be written as

$$\mathrm{AUC}_\alpha^\beta = (Area_A + Area_B) - Area_B \tag{42}$$

$$= \int_{S_{\bar{D}}(S_D^{-1}(1-\alpha))}^{\beta} S_D(S_{\bar{D}}^{-1}(t))\mathrm{d}t - (1-\alpha)\left(\beta - S_{\bar{D}}\left(S_D^{-1}(1-\alpha)\right)\right) \tag{43}$$

$\downarrow$ See following proof.

$$= P\left[Y_D > Y_{\bar{D}}, Y_D \leq S_D^{-1}(1-\alpha), Y_{\bar{D}} \geq S_{\bar{D}}^{-1}(\beta)\right] \tag{44}$$

$$= P\left[S_D^{-1}(1-\alpha) \geq Y_D > Y_{\bar{D}} \geq S_{\bar{D}}^{-1}(\beta)\right]. \tag{45}$$

Our method uses two special cases of $\mathrm{AUC}_\alpha^\beta$: The bottom-left ($AUC_{high}$, green) and top-right ($\mathrm{AUC}_{low}$, red) part of the AUC, shown in Supplementary Figure 18.

Proof

With

$$y_0 = S_{\bar{D}}^{-1}(t_0), \qquad y_1 = S_{\bar{D}}^{-1}(t_1), \tag{46}$$

we have

$$\int_{t_1}^{t_0} \mathrm{ROC}(t)dt = \int_{t_1}^{t_0} S_D(\underbrace{S_{\bar{D}}^{-1}(t)}_{=y})dt = \int_{y_1}^{y_0} S_D(y)S'_{\bar{D}}(y)dy = \int_{y_0}^{y_1} S_D(y)f_{\bar{D}}(y)dy\,. \tag{47}$$

From

$$S_D(y) = \int_y^\infty f_D(u)du = \int_y^{y_1} f_D(u)du + \int_{y_1}^\infty f_D(u)du = \int_y^{y_1} f_D(u)du + S_D(y_1) \tag{48}$$

we obtain

$$\int_{t_1}^{t_0} \mathrm{ROC}(t)dt = \int_{y_0}^{y_1} S_D(y)f_{\bar{D}}(y)dy \tag{49}$$

$$= \int_{y_0}^{y_1} \left[\int_y^{y_1} f_D(u)du + S_D(y_1)\right] f_{\bar{D}}(y)dy \tag{50}$$

$$= \int_{y_0}^{y_1} \int_y^{y_1} f_D(u)du f_{\bar{D}}(y)dy + S_D(y_1)\int_{y_0}^{y_1} f_{\bar{D}}(y)dy$$

$$= \int_{y_0}^{y_1} \int_y^{y_1} f_D(u)du f_{\bar{D}}(y)dy + S_D(y_1)\left(S_{\bar{D}}(y_0) - S_{\bar{D}}(y_1)\right)$$

$$= P[y_1 \geq Y_D > Y_{\bar{D}} \geq y_0] + S_D(y_1)\left(t_0 - t_1\right)\,. \tag{51}$$

For our application, use

$$t_0 = \beta\,, \quad y_0 = S_{\bar{D}}^{-1}(\beta)\,, \quad t_1 = S_{\bar{D}}(S_D^{-1}(1-\alpha))\,, \quad y_1 = S_D^{-1}(1-\alpha)\,, \quad S_D(y_1) = (1-\alpha)\,, \tag{52}$$

to obtain the two-way partial AUC

$$\mathrm{AUC}_\alpha^\beta = \int_{S_{\bar{D}}\left(S_D^{-1}(1-\alpha)\right)}^{\beta} \mathrm{ROC}(t)\mathrm{d}t - (1-\alpha)\left(\beta - S_{\bar{D}}\left(S_D^{-1}(1-\alpha)\right)\right)\,. \tag{53}$$

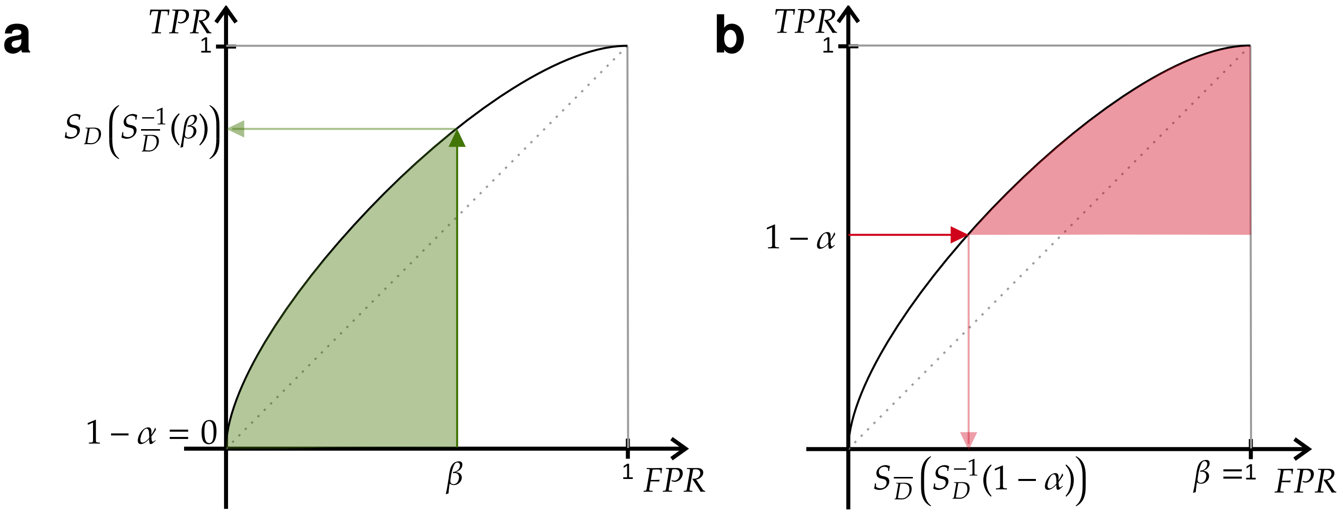

Supplementary Figure 18 *ROC curves showing two-way partial AUCs corresponding to* $marker^{HIGH}$ *and* $marker^{LOW}$ *samples used in our restriction method.*
Partial AUCs using the nomenclature from Supplementary Figure 17. **(a)** Two-way partial AUC with $\alpha = 1$ and a maximum false positive rate β. β corresponds to a specific threshold through $c = S_{\bar{D}}^{-1}(\beta)$. Therefore this green area corresponds to the AUC calculated for the complete ROC curve including $marker^{HIGH}$ samples. **(b)** Two-way partial AUC with $\beta = 1$ and a minimum true positive rate of $1-\alpha$. $1-\alpha$ corresponds to a specific threshold through $c = S_D^{-1}(1-\alpha)$. Therefore this red area corresponds to the AUC calculated for the complete ROC curve including $marker^{LOW}$ samples.

4.2.1 AUC_{high}

The left part of the area under the curve up to a false positive rate β is identical to the pAUC described earlier (Equation (31))

$$\mathrm{AUC}_{high}(\beta) = \mathrm{AUC}^{\beta}_{\alpha\equiv 1} = \tag{54}$$

$$\Big\downarrow \text{Equation (43)}$$

$$= \int_{S_{\bar{D}}\left(S_D^{-1}(1-\underset{\equiv 1}{\alpha})\right)}^{\beta} \mathrm{ROC}(t)\mathrm{d}t - (1-\underset{\equiv 1}{\alpha})\left(\beta - S_{\bar{D}}\left(S_D^{-1}(1-\underset{\equiv 1}{\alpha})\right)\right) \tag{55}$$

$$= \int_{S_{\bar{D}}\left(S_D^{-1}(0)\right)}^{\beta} \mathrm{ROC}(t)\mathrm{d}t \tag{56}$$

$$\Big\downarrow \text{Equation (5)}$$

$$= \int_{S_{\bar{D}}(\infty)}^{\beta} \mathrm{ROC}(t)\mathrm{d}t \tag{57}$$

$$= \int_{0}^{\beta} \mathrm{ROC}(t)\mathrm{d}t \overset{Equation\ (31)}{=} pAUC(\beta). \tag{58}$$

$$\text{AUC}_{high}(\beta) =$$

$\downarrow$ Equation (44)

$$= P\left[Y_D > Y_{\overline{D}}, Y_D \le S_D^{-1}(1 - \underset{\equiv 1}{\alpha}), Y_{\overline{D}} \ge S_{\overline{D}}^{-1}(\beta)\right] \tag{59}$$

$$= P\left[Y_D > Y_{\overline{D}}, Y_D \le S_D^{-1}(0), Y_{\overline{D}} \ge S_{\overline{D}}^{-1}(\beta)\right] \tag{60}$$

$$= P\left[Y_D > Y_{\overline{D}}, Y_D \le \infty, Y_{\overline{D}} \ge S_{\overline{D}}^{-1}(\beta)\right] \tag{61}$$

$$= P\left[Y_D > Y_{\overline{D}}, Y_{\overline{D}} \ge S_{\overline{D}}^{-1}(\beta)\right] \tag{62}$$

$\downarrow$ Equation (39) and $t_0 := \beta$

$$= pAUC(\beta). \tag{63}$$

4.2.2 AUC_{low}

The right part of the area under the curve with at least a true positive rate of $1 - \alpha$

$$\text{AUC}_{low}(\alpha) = \text{AUC}_{\alpha}^{\beta \equiv 1} = \tag{64}$$

$\downarrow$ Equation (43)

$$= \int_{S_{\overline{D}}\left(S_D^{-1}(1-\alpha)\right)}^{\underset{\equiv 1}{\beta}} \text{ROC}(t)\mathrm{d}t - (1-\alpha)\left(\underset{\equiv 1}{\beta} - S_{\overline{D}}\left(S_D^{-1}(1-\alpha)\right)\right) \tag{65}$$

$$= \int_{S_{\overline{D}}\left(S_D^{-1}(1-\alpha)\right)}^{1} \text{ROC}(t)\mathrm{d}t - (1-\alpha)\left(1 - S_{\overline{D}}\left(S_D^{-1}(1-\alpha)\right)\right). \tag{66}$$

$$\text{AUC}_{low}(\alpha) =$$

$\downarrow$ Equation (44)

$$= P\left[Y_D > Y_{\overline{D}}, Y_D \le S_D^{-1}(1-\alpha), Y_{\overline{D}} \ge S_{\overline{D}}^{-1}(\underset{\equiv 1}{\beta})\right] \tag{67}$$

$\downarrow$ Equation (5)

$$= P\left[Y_D > Y_{\overline{D}}, Y_D \le S_D^{-1}(1-\alpha), Y_{\overline{D}} \ge -\infty\right] \tag{68}$$

$$= P\left[Y_D > Y_{\overline{D}}, Y_D \le S_D^{-1}(1-\alpha)\right] \tag{69}$$

$$= P\left[Y_D \le S_D^{-1}(1-\alpha)\right] P\left[Y_D > Y_{\overline{D}} | Y_D \le S_D^{-1}(1-\alpha)\right] \tag{70}$$

$$= (1-\alpha) \cdot P\left[Y_D > Y_{\overline{D}} | Y_D \le S_D^{-1}(1-\alpha)\right]. \tag{71}$$

4.3 Restricted sample space

4.3.1 ROC for restricted sample space

We restrict our sample space to

$$y_0 \leq Y_X \leq y_1 \qquad \text{for both} \quad X \in \{D, \overline{D}\}\,. \tag{72}$$

This restriction leads to modified probability density functions

$$\tilde{f}_X(y) = \begin{cases} \frac{1}{Z_X} f_X(y) & \text{for } y \in [y_0, y_1]\,, \\ 0 & \text{otherwise}\,. \end{cases} \tag{73}$$

Throughout, explicit dependence on y_0, y_1 is omitted to increase readability but always implied. The (y-independent) constants

$$Z_X = \int_{y_0}^{y_1} f_X(y)dy = S_X(y_0) - S_X(y_1) = P[y_1 \geq Y_X \geq y_0] \tag{74}$$

ensure proper normalization of the densities,

$$\int_{-\infty}^{\infty} \tilde{f}_X(y)dy = \int_{y_0}^{y_1} \tilde{f}_X(y)dy = \frac{1}{Z_X}\int_{y_0}^{y_1} f_X(y)dy = 1\,. \tag{75}$$

This means that $\tilde{f}_X$ can be interpreted as a conditional probability density function for Y_X, conditioned on the restriction $y_0 \leq Y_X \leq y_1$. For quantities defined on the restricted space we use $\widetilde{(\cdot)}$.

On the restricted space, true and false positive rates can be determined from the corresponding densities $\tilde{f}_D$ and $\tilde{f}_{\overline{D}}$ in the usual way,

$$\widetilde{S}_X(y) = \int_y^{\infty} \tilde{f}_X(u)du = \begin{cases} 1 & \text{for } y < y_0\,, \\ \int_y^{y_1} \tilde{f}_X(u)du & \text{for } y_0 \leq y \leq y_1\,, \\ 0 & \text{for } y_1 < y\,. \end{cases} \tag{76}$$

For the non-trivial case $y_0 \leq y \leq y_1$, we therefore obtain

$$\begin{aligned} \widetilde{S}_X(y) &= \int_y^{y_1} \tilde{f}_X(u)du = \frac{1}{Z_X}\int_y^{y_1} f_X(u)du = \frac{S_X(y) - S_X(y_1)}{S_X(y_0) - S_X(y_1)} = \frac{P[y \leq Y_X \leq y_1]}{P[y_0 \leq Y_X \leq y_1]} \\ &= \frac{P[y_0 \leq y \leq Y_X \leq y_1]}{P[y_0 \leq Y_X \leq y_1]} = \frac{P[y \leq Y_X\,,\, y_0 \leq Y_X \leq y_1]}{P[y_0 \leq Y_X \leq y_1]} = P[y \leq Y_X \,|\, y_0 \leq Y_X \leq y_1]\,, \end{aligned} \tag{77}$$

where the probabilities $P[.]$ are defined w.r.t the original (unrestricted) density functions f_X. Equation (77) can be rewritten as

$$\widetilde{S}_X(y) = \frac{1}{S_X(y_0) - S_X(y_1)} S_X(y) - \frac{S_X(y_1)}{S_X(y_0) - S_X(y_1)} = \gamma_X S_X(y) - \delta_X \tag{78}$$

with (y-independent) constants

$$\gamma_X = \frac{1}{Z_X} = \frac{1}{S_X(y_0) - S_X(y_1)} \qquad \text{and} \qquad \delta_X = \frac{S_X(y_1)}{S_X(y_0) - S_X(y_1)} = \gamma_X S_X(y_1)\,. \tag{79}$$

This establishes a one-to-one map between the unrestricted rates $S_X(y) \in [S_X(y_1), S_X(y_0)]$ and their restricted counterparts $\widetilde{S}_X(y) \in [0, 1]$, through a simple shift (by δ_X) and rescaling (with γ_X).

From the rate functions $\widetilde{S}_X$, we obtain the corresponding ROC function on the restricted space,

$$\mathrm{rROC}(t) = \widetilde{S}_D\left(\widetilde{S}_{\overline{D}}^{-1}(t)\right), \quad t \in [0,1]. \tag{80}$$

Note that the interval $[0,1]$ for t corresponds to thresholds $y \in [y_0, y_1]$. As the identity Equation (78) applies to both $X \in \{D, \overline{D}\}$, we have a one-to-one map between points on the original ROC curve obtained from thresholds $y \in [y_0, y_1]$ and points on the rROC curve for the restricted densities:

$$\begin{pmatrix} S_{\overline{D}}(y) \\ S_D(y) \end{pmatrix} \quad \mapsto \quad \begin{pmatrix} \widetilde{S}_{\overline{D}}(y) \\ \widetilde{S}_D(y) \end{pmatrix} = \begin{pmatrix} \gamma_{\overline{D}} S_{\overline{D}}(y) \\ \gamma_D S_D(y) \end{pmatrix} - \begin{pmatrix} \delta_{\overline{D}} \\ \delta_D \end{pmatrix}. \tag{81}$$

In particular

$$\begin{pmatrix} S_{\overline{D}}(y_0) \\ S_D(y_0) \end{pmatrix} \quad \mapsto \quad \begin{pmatrix} \widetilde{S}_{\overline{D}}(y_0) \\ \widetilde{S}_D(y_0) \end{pmatrix} = \begin{pmatrix} 1 \\ 1 \end{pmatrix}, \tag{82}$$

$$\begin{pmatrix} S_{\overline{D}}(y_1) \\ S_D(y_1) \end{pmatrix} \quad \mapsto \quad \begin{pmatrix} \widetilde{S}_{\overline{D}}(y_1) \\ \widetilde{S}_D(y_1) \end{pmatrix} = \begin{pmatrix} 0 \\ 0 \end{pmatrix}. \tag{83}$$

This means that the rROC curve for the restricted sample space can be obtained from the original ROC curve by simply cutting out a rectangle corresponding to the intervals $[S_{\overline{D}}(y_1), S_{\overline{D}}(y_0)]$ and $[S_D(y_1), S_D(y_0)]$ and rescaling both axes to $[0,1]$ afterwards.

4.3.2 AUC for restricted sample space

Since we have (see Equation (77))

$$\widetilde{S}_D(y) = P[y \le Y_D \,|\, y_0 \le Y_D \le y_1], \tag{84}$$

$$\widetilde{S}_{\overline{D}}(y) = P[y \le Y_{\overline{D}} \,|\, y_0 \le Y_{\overline{D}} \le y_1], \tag{85}$$

the area under the rROC curve is given by

$$\mathrm{rAUC} = P[Y_{\overline{D}} \le Y_D \,|\, y_0 \le Y \le y_1]. \tag{86}$$

By definition, we have

$$\begin{aligned} P[y_0 \le Y \le y_1] &= P[y_0 \le Y_D \le y_1] P[y_0 \le Y_{\overline{D}} \le y_1] = Z_D Z_{\overline{D}} \\ &= (S_D(y_0) - S_D(y_1)) \left(S_{\overline{D}}(y_0) - S_{\overline{D}}(y_1)\right). \end{aligned} \tag{87}$$

For rAUC we then obtain

$$\mathrm{rAUC} = \frac{P[Y_{\overline{D}} \le Y_D \,,\, y_0 \le Y \le y_1]}{P[y_0 \le Y \le y_1]} \tag{88}$$

$$= \frac{P[y_0 \le Y_{\overline{D}} \le Y_D \le y_1]}{Z_D Z_{\overline{D}}} \tag{89}$$

$$= \frac{P[y_0 \le Y_{\overline{D}} \le Y_D \le y_1]}{(S_D(y_0) - S_D(y_1)) \left(S_{\overline{D}}(y_0) - S_{\overline{D}}(y_1)\right)}. \tag{90}$$

4.3.3 Restricted AUC in terms of true and false positive rates

We can write Equation (86) in terms of a minimum true positive rate $1 - \alpha$ and a maximum false positive rate β by defining

$$y_0 := S_{\bar{D}}^{-1}(\beta)\,, \qquad y_1 := S_D^{-1}(1-\alpha), \tag{91}$$

to obtain

$$\mathrm{rAUC}_\alpha^\beta := P[Y_D > Y_{\bar{D}} \,|\, S_{\bar{D}}^{-1}(\beta) \le Y \le S_D^{-1}(1-\alpha)], \tag{92}$$

where $S_{\bar{D}}^{-1}(\beta) \le Y \le S_D^{-1}(1-\alpha)$ is the condition that all values (Y_D and $Y_{\bar{D}}$) are between $S_{\bar{D}}^{-1}(\beta)$ and $S_D^{-1}(1-\alpha)$.
With Equation (87) we get a scaling factor

$$P[y_0 \le Y \le y_1] = \Big(S_D(S_{\bar{D}}^{-1}(\beta)) - (1-\alpha)\Big)\Big(\beta - S_{\bar{D}}(S_D^{-1}(1-\alpha))\Big) = \text{scaling}_\alpha^\beta\,. \tag{93}$$

This means that with Equation (45) and Equation (90), the restricted AUC can *also* be obtained from the original two-way partial AUC by dividing by this scaling factor:

$$\mathrm{rAUC}_\alpha^\beta = \frac{\mathrm{AUC}_\alpha^\beta}{\text{scaling}_\alpha^\beta}\,. \tag{94}$$

This is directly visible from Supplementary Figure 17 where a rectangle spanning across area A has a width of

$$w = \beta - S_{\bar{D}}(S_D^{-1}(1-\alpha)), \tag{95}$$

and a height of

$$h = S_D(S_{\bar{D}}^{-1}(\beta)) - (1-\alpha) = \mathrm{ROC}(\beta) - (1-\alpha), \tag{96}$$

which results in the scaling factor scaling$_\alpha^\beta$.

4.4 Restriction

Until now, $\mathrm{rAUC}_{\alpha}^{\beta}$ is defined in terms of maximum false positive rate β and minimum true positive rate $1-\alpha$. Alternatively, we here introduce a "restriction" $r \in \mathbb{R}$ which splits the data into high and low parts where $\alpha := 1 - S_D(r)$ and $\beta := S_{\bar{D}}(r)$, respectively.

4.4.1 $\mathrm{rAUC}_{high}(r)$

$$\mathrm{rAUC}_{high}(r) := \mathrm{rAUC}_{\alpha\equiv 1}^{\beta\equiv S_{\bar{D}}(r)} = \tag{97}$$

$\downarrow$ Equation (94)

$$= \mathrm{AUC}_{high}(S_{\bar{D}}(r)) \cdot \frac{1}{S_{\bar{D}}(r) - S_{\bar{D}}\left(S_D^{-1}(1-1)\right)} \cdot \frac{1}{S_D\left(S_{\bar{D}}^{-1}\left(S_{\bar{D}}(r)\right)\right) - (1-1)} \tag{98}$$

$\downarrow$ Equation (5): $S_D^{-1}(0) = \infty$ and $S_{\bar{D}}(\infty) = 0$

$$= \mathrm{AUC}_{high}(S_{\bar{D}}(r)) \cdot \frac{1}{S_{\bar{D}}(r)} \cdot \frac{1}{S_D(r)}. \tag{99}$$

4.4.2 $\mathrm{rAUC}_{low}(r)$

$$\mathrm{rAUC}_{low}(r) := \mathrm{rAUC}_{\alpha\equiv 1-S_D(r)}^{\beta\equiv 1} = \tag{100}$$

$\downarrow$ Equation (94)

$$= \mathrm{AUC}_{low}(1 - S_D(r)) \cdot \frac{1}{1 - S_{\bar{D}}\left(S_D^{-1}\left(1-(1-S_D(r))\right)\right)} \cdot \frac{1}{S_D\left(S_{\bar{D}}^{-1}(1)\right) - \left(1-(1-S_D(r))\right)} \tag{101}$$

$\downarrow$ Equation (5): $S_{\bar{D}}^{-1}(1) = -\infty$ and $S_D(-\infty) = 1$

$$= \mathrm{AUC}_{low}(1 - S_D(r)) \cdot \frac{1}{1 - S_{\bar{D}}\left(S_D^{-1}\left(S_D(r)\right)\right)} \cdot \frac{1}{1-\left(1-(1-S_D(r))\right)} \tag{102}$$

$$= \mathrm{AUC}_{low}(1 - S_D(r)) \cdot \frac{1}{1 - S_{\bar{D}}(r)} \cdot \frac{1}{1 - S_D(r)}. \tag{103}$$

Supplementary Figure 19

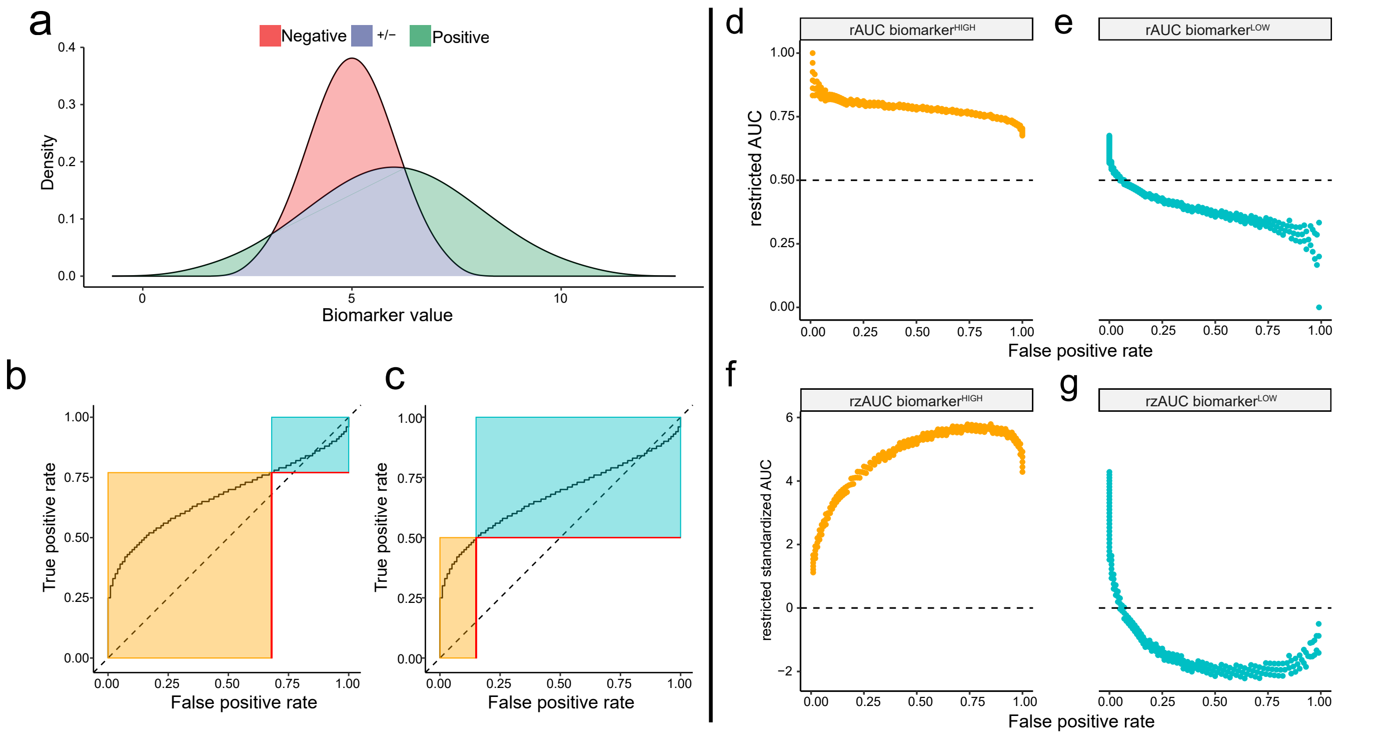

Supplementary Figure 19 *Densities, ROC curves, restricted AUCs and restricted standardized AUCs.* **(a)** A simulated distribution of 100 negative $\mathcal{N}(5, 1)$ and 100 positive $\mathcal{N}(6, 2)$ samples. **(b)** The corresponding right-skewed complete ROC curve shows two possible restriction levels, which illustrates that a restriction can be made at every sample value. Each restriction divides the samples into sets of marker^{HIGH} (orange) and marker^{LOW} (blue) samples. **(c)** See (b). **(d)** Restricted AUC (rAUC) of marker^{HIGH} samples against the FPR calculated for every possible restriction. **(e)** Restricted AUC (rAUC) of marker^{LOW} samples against the FPR calculated for every possible restriction. **(f)** Plots of the restricted standardized AUC (rzAUC) against the FPR calculated for every possible restriction on marker^{HIGH} samples. **(g)** Plots of the restricted standardized AUC (rzAUC) against the FPR calculated for every possible restriction on marker^{LOW} samples.

Supplementary Figure 20

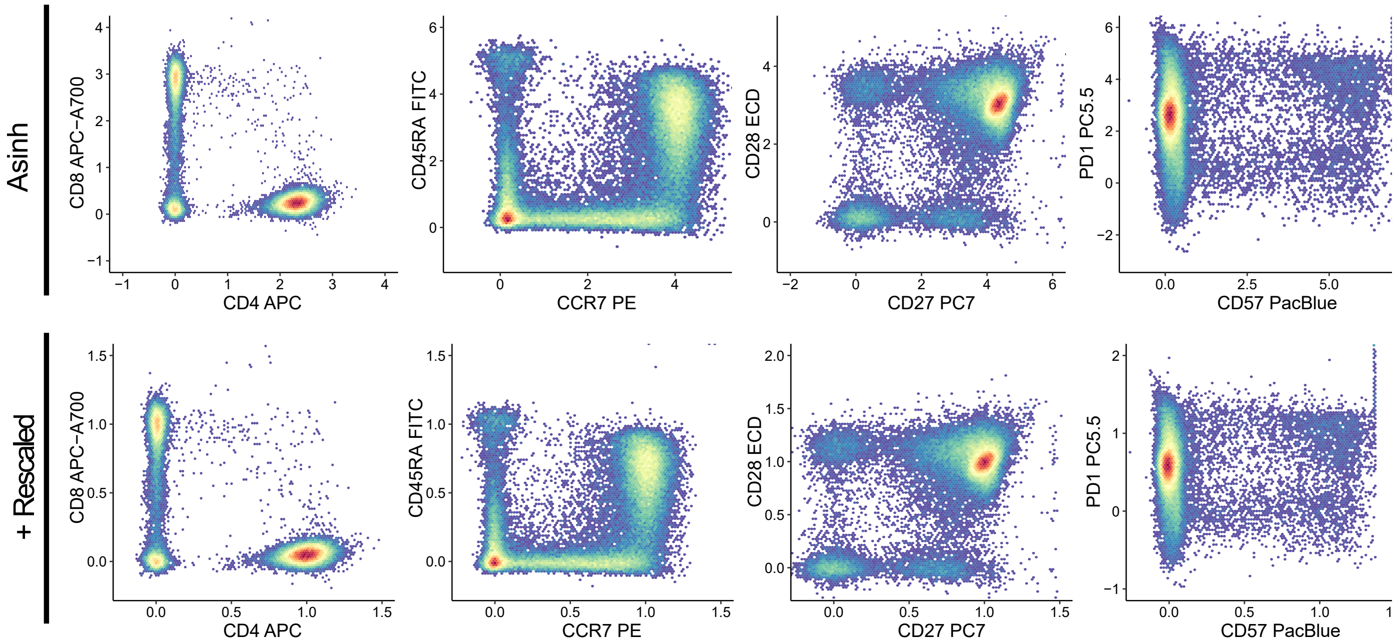

Supplementary Figure 20 *Pre-processing of flow cytometry data.*

Following a conventional workflow, an experienced, blinded operator performed sample-wise manual recompensation of flow cytometry data using Kaluza software. Cell antigen-wise asinh-transformations and rescaling were then applied. Here, we illustrate our data pre-processing with a single sample. The upper row shows all events after cell antigen-wise asinh transformation. The lower row shows the same sample after rescaling.

Supplementary Figure 21

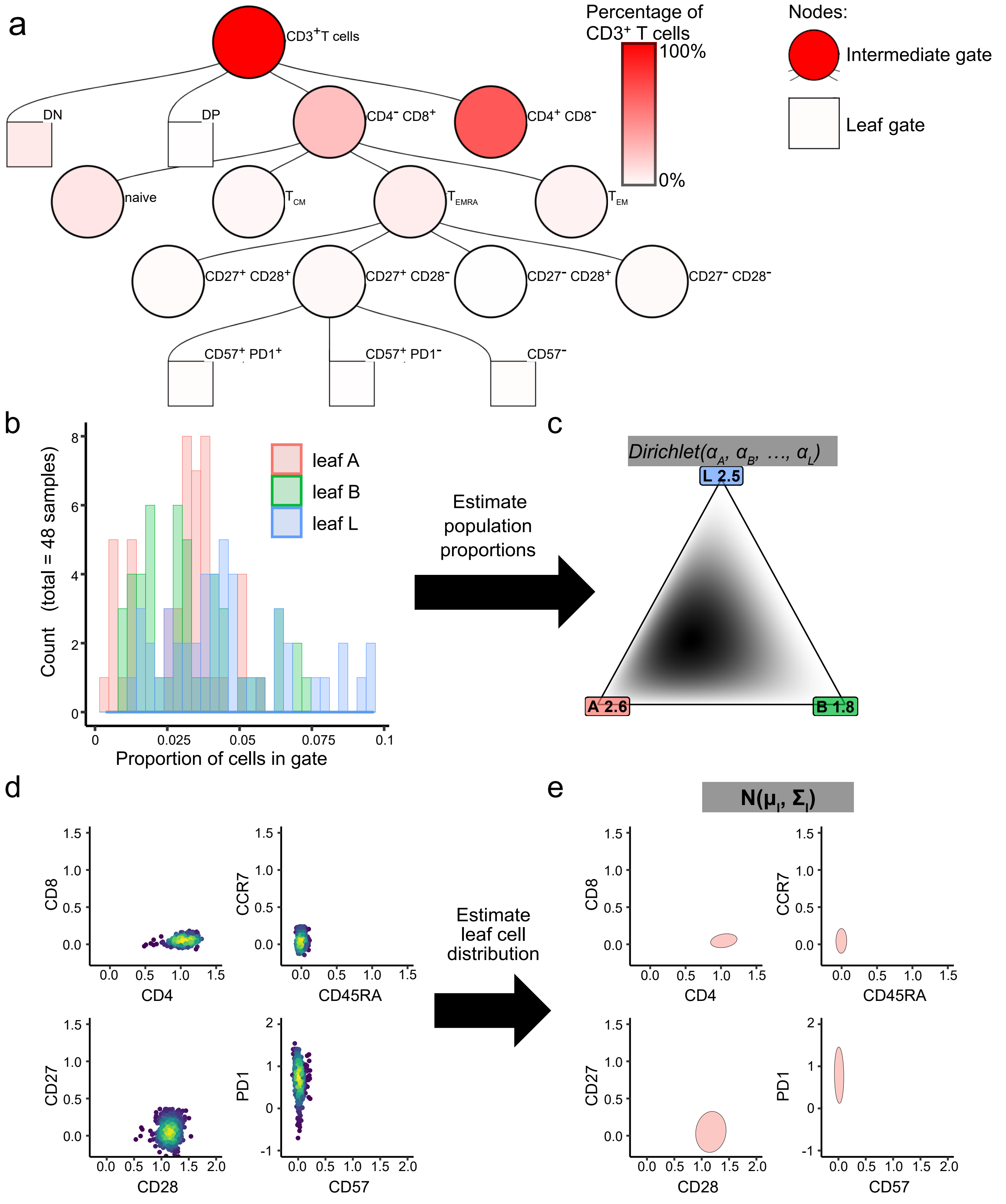

Supplementary Figure 21 *Estimating cell population parameters as a basis for synthesizing realistic flow cytometry data.*

Supplementary Figure 21 *Estimating cell population parameters as a basis for synthesizing realistic flow cytometry data.* **(a)** A hierarchical gating tree used to classify peripheral blood T cell subsets. Within this hierarchy, intermediate gates (circles) contain the cells that are categorized into end-nodes by leaf gates (rectangles). Typical frequencies of cells in all gates were estimated from 48 samples, which represented 6 replicate stainings from 8 healthy donors. Gates are coloured according to the percentage of all cells they encompass. **(b)** A histogram showing the number of cells from all samples in three arbitrarily selected leaf gates. Leaf A (red) is the $CD27^{-}$ $CD28^{+}$ $CD57^{-}$ $CD4^{+}$ T_{EM} cell gate. Leaf B (green) is the $CD27^{+}$ $CD28^{-}$ $CD57^{-}$ $CD8^{+}$ T_{EMRA} cell gate. Leaf L (blue) is the $CD27^{-}$ $CD28^{+}$ $CD57^{-}$ $CD4^{+}$ T_{CM} cell gate. **(c)** A plot showing the corresponding density of the Dirichlet distribution for example leaf gates A, B and L. Black shading indicates higher probabilities. **(d)** 2-dimensional scatter plots showing the distribution of cell-surface protein expression (i.e. cell antigens) used to define cells that fall within leaf A. Higher cell densities are indicated with yellow shading. **(e)** Expression values for all 8 cell antigens were used to estimate a multivariate normal distribution for every leaf. The resulting leaf cell distribution for leaf A is depicted as shaded areas representing normal distributions ± 1 standard deviation.

Supplementary Figure 22

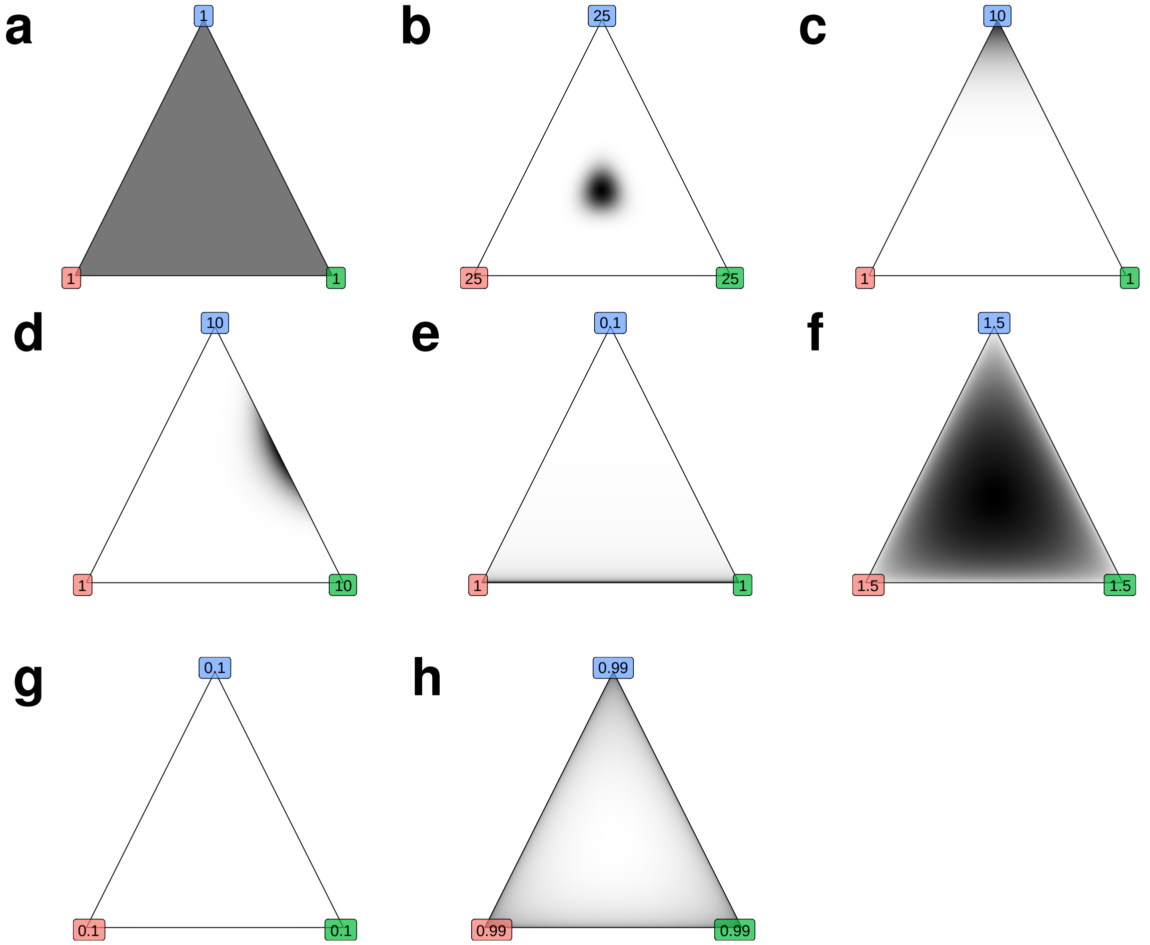

Supplementary Figure 22 *Dirichlet probability density examples for K=3.*

Each subfigure shows the probability density for different parameters α_1, α_2 and α_3 from a Dirichlet distribution $p(\mathbf{p}) \sim Dir(\alpha_1, \alpha_2, \alpha_3)$. Parameter values are shown at the corners and also in Supplementary Table 2.

Supplementary Table 2

Subfigure	α_1	α_2	α_3	m_1	m_2	m_3	var_1	var_2	var_3	cov_{12}	cov_{13}	cov_{23}
a	1.00	1.00	1.00	0.33	0.33	0.33	0.06	0.06	0.06	-0.03	-0.03	-0.03
b	25.00	25.00	25.00	0.33	0.33	0.33	0.00	0.00	0.00	-0.00	-0.00	-0.00
c	1.00	1.00	10.00	0.08	0.08	0.83	0.01	0.01	0.01	-0.00	-0.01	-0.01
d	1.00	10.00	10.00	0.05	0.48	0.48	0.00	0.01	0.01	-0.00	-0.00	-0.01
e	1.00	1.00	0.10	0.48	0.48	0.05	0.08	0.08	0.01	-0.07	-0.01	-0.01
f	1.50	1.50	1.50	0.33	0.33	0.33	0.04	0.04	0.04	-0.02	-0.02	-0.02
g	0.10	0.10	0.10	0.33	0.33	0.33	0.17	0.17	0.17	-0.09	-0.09	-0.09
h	0.99	0.99	0.99	0.33	0.33	0.33	0.06	0.06	0.06	-0.03	-0.03	-0.03

Supplementary Table 2 *Parameters, means, variances and covariances for probability densities shown in Supplementary Figure 22.*
The table shows the parameters α_1, α_2 and α_3 from a Dirichlet distribution. Each row corresponds to the respective subfigure in Supplementary Figure 22. m_1, m_2 and m_3 are the means for each of the parameters $(m_i = E[p_i] = \frac{\alpha_i}{s}$ for $s = \sum_k \alpha_k)$. var_1, var_2 and var_3 are the variances for each of the parameters $(var_i = Var[p_i] = \frac{\alpha_i(s-\alpha_i)}{s^2(s+1)})$. cov_{12}, cov_{13} and cov_{23} are the covariances for each of the parameters $(cov_{ij} = Cov[p_i, p_j] = \frac{-\alpha_i\alpha_j}{(s^2(s+1))})$.

Supplementary Figure 23

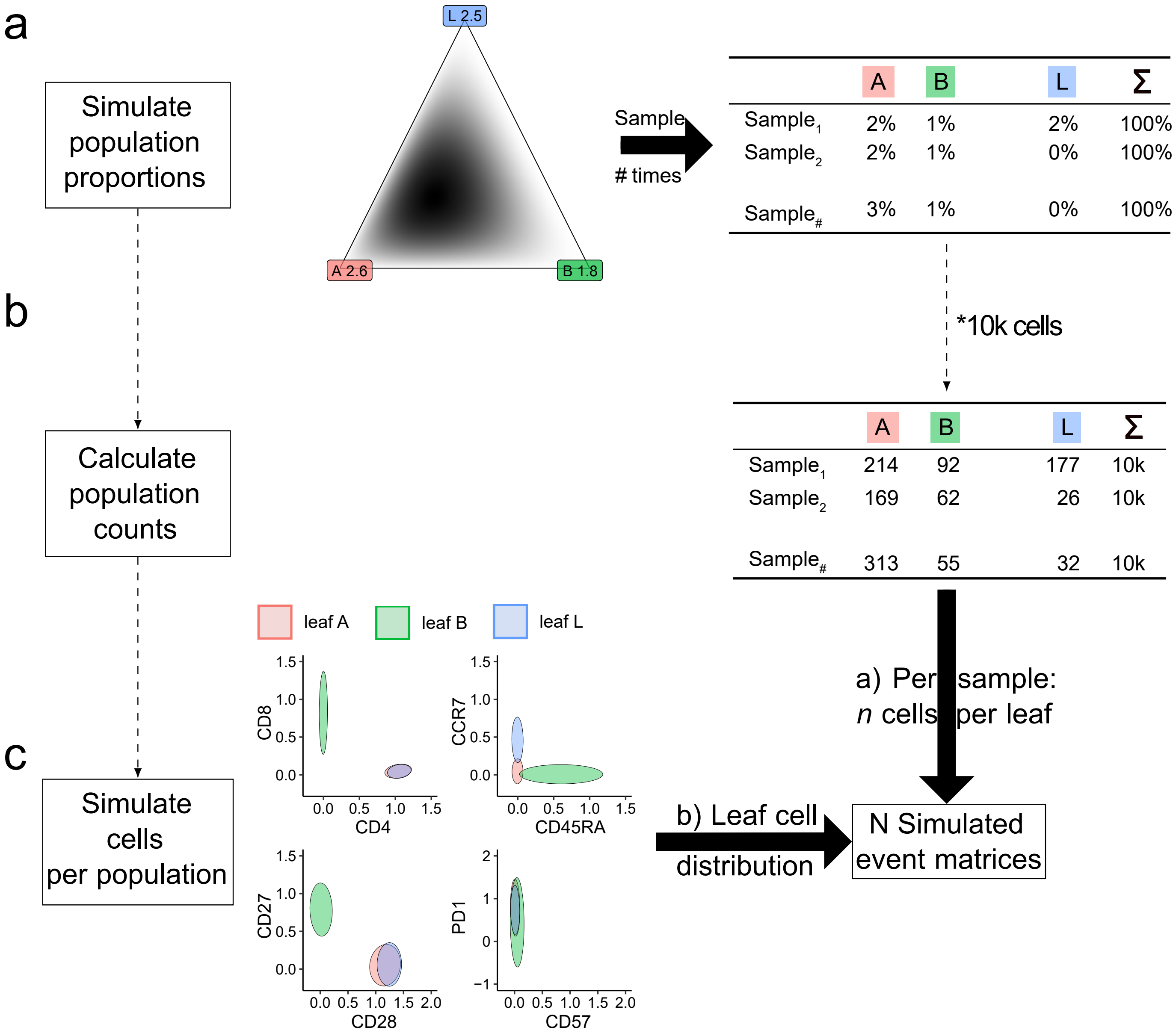

Supplementary Figure 23 *Procedure for generating realistic flow cytometry data.*

There are three steps to synthesizing new flow cytometry samples. **(a)** First, we generate cell population proportions for N new samples, where the percentage of cells in each gate is drawn from the previously established Dirichlet distribution. **(b)** Second, we calculate cell counts for all leaf gates by multiplying the simulated percentages by the desired overall number of cells; in this example, 10,000 total cells. **(c)** Third, we draw the required number of cells per leaf gate from its previously estimated multivariate normal distribution. This procedure is repeated for N newly synthetic samples. Thus, we obtain an event matrix of 10,000 cells for each of the N samples.

References

[1] Bamber, D. (1975). The area above the ordinal dominance graph and the area below the receiver operating characteristic graph. In Journal of Mathematical Psychology (Vol. 12, Issue 4, pp. 387-415). Elsevier BV. https://doi.org/10.1016/0022-2496(75)90001-2

[2] Dodd, L.E. (2001). Regression methods for areas and partial areas under the ROC curve. Ph.D. thesis, University of Washington

[3] Glehr G., Riquelme P., Yang Zhou J., Cordero L., Schilling H-L., Kapinsky M., Schlitt HJ., Geissler EK., Burkhardt R., Schmidt B., Haferkamp S., Hutchinson JA. & Kronenberg K. (2022). External validation of biomarkers for immune-related adverse events after immune checkpoint inhibition. In Front. Immunol. 13:1011040. doi: 10.3389/fimmu.2022.1011040

[4] Hanley, J. A., & McNeil, B. J. (1982). The meaning and use of the area under a receiver operating characteristic (ROC) curve. In Radiology (Vol. 143, Issue 1, pp. 29-36). Radiological Society of North America (RSNA). https://doi.org/10.1148/radiology.143.1.7063747

[5] Harel, M., Ortenberg, R., Varanasi, S. K., Mangalhara, K. C., Mardamshina, M., Markovits, E., ... & Geiger, T. (2019). Proteomics of melanoma response to immunotherapy reveals mitochondrial dependence. Cell, 179(1), 236-250.

[6] Lee, K. A., Thomas, A. M., Bolte, L. A., Björk, J. R., de Ruijter, L. K., Armanini, F., ... & Segata, N. (2022). Cross-cohort gut microbiome associations with immune checkpoint inhibitor response in advanced melanoma. Nature Medicine, 28(3), 535-544.

[7] Lozano, A. X., Chaudhuri, A. A., Nene, A., Bacchiocchi, A., Earland, N., Vesely, M. D., ... & Newman, A. M. (2022). T cell characteristics associated with toxicity to immune checkpoint blockade in patients with melanoma. Nature Medicine, 28(2), 353-362.

[8] Sullivan Pepe, M. (2004). The statistical evaluation of medical tests for classification and prediction. Oxford University Press.

[9] Yang, H., Lu, K., Lyu, X., & Hu, F. (2017). Two-way partial AUC and its properties. In Statistical Methods in Medical Research (Vol. 28, Issue 1, pp. 184-195). SAGE Publications. https://doi.org/10.1177/0962280217718866

[10] Z. Yang, Q. Xu, S. Bao, Y. He, X. Cao and Q. Huang (2022). Optimizing Two-way Partial AUC with an End-to-end Framework". In IEEE Transactions on Pattern Analysis and Machine Intelligence. doi: 10.1109/TPAMI.2022.3185311

[11] Zhang, Z., Wang, Z. X., Chen, Y. X., Wu, H. X., Yin, L., Zhao, Q., ... & Xu, R. H. (2022). Integrated analysis of single-cell and bulk RNA sequencing data reveals a pan-cancer stemness signature predicting immunotherapy response. Genome Medicine, 14(1), 45.
