# Supplementary material for: Restricting datasets to classifiable samples augments discovery of immune disease biomarkers
Source: Nat Commun. 2024 Jun 26;15:5417. doi: 10.1038/s41467-024-49094-3 (PMC11208602; doi:10.1038/s41467-024-49094-3)
Supplement: Supplementary file 3 — Description of Additional Supplementary Files [file 41467_2024_49094_MOESM3_ESM.pdf]

Description of Additional Supplementary Files

Supplementary Movie 1: Restriction method applied to simulated distributions with varying mean of the positive population (GIF).

We present simulated examples of biomarker normal distributions in two classes varying the positive population's mean. Positive and negative populations are intended to represent sets of patients with different clinical outcomes with varying effect sizes. The distribution of values from the positive (i.e. diseased) class is coloured green and values from the negative (i.e. control) class is coloured red; the overlapping density areas are coloured purple. For each example, we present the following 5 plots: (a) the positive and negative class densities; (b) the complete receiver operator characteristic (ROC) curve; (c) a plot of biomarker values against FPR; (d) a plot of rzAUC calculated for $\text{marker}^{\text{HIGH}}$ (orange) and $\text{marker}^{\text{LOW}}$ (blue) samples at all FPR values. (e) The restricted (blue) and unrestricted (red, "global") AUC against the mean of the positive population. In plots (a)-(d), red lines indicate the optimal restriction as a biomarker value or FPR value. The GIF shows the five plots when the mean of the positive population varies from $\mathcal{N}(5,2)$ to $\mathcal{N}(9,2)$ and the negative population remains $\mathcal{N}(5,1)$. 100 samples are drawn from every population.

Supplementary Movie 2: Restriction method applied to simulated distributions with varying variance of the positive population (GIF).

We present simulated examples of biomarker normal distributions in two classes varying the positive population's variance. Positive and negative populations are intended to represent sets of patients with different clinical outcomes. The distribution of values from the positive (i.e. diseased) class is coloured green and values from the negative (i.e. control) class is coloured red; the overlapping density areas are coloured purple. For each example, we present the following 5 plots: (a) the positive and negative class densities; (b) the complete receiver operator characteristic (ROC) curve; (c) a plot of biomarker values against FPR; (d) a plot of rzAUC calculated for $\text{marker}^{\text{HIGH}}$ (orange) and $\text{marker}^{\text{LOW}}$ (blue) samples at all FPR values. (e) The restricted (blue) and unrestricted (red, "global") AUC against the standard deviation of the positive population. In plots (a)-(d), red lines indicate the optimal restriction as a biomarker value or FPR value. The GIF shows the five plots when the variance of the positive population varies from $\mathcal{N}(7,1)$ to $\mathcal{N}(7,25)$ and the negative population remains $\mathcal{N}(5,1)$. 100 samples are drawn from every population.

Supplementary Movie 3: Restricted AUC, correspondence of scaling factor and restriction (GIF).

We present a simulated example of biomarker distributions in two classes, which are intended to represent sets of patients with different clinical outcomes. We created a GIF for all possible restrictions and show the following 6 plots for each restriction. (a) The distribution of values from the positive (i.e. diseased, n=100) class are coloured green and values from the negative (i.e. control, n=100) class are coloured red; the overlapping density areas are coloured in purple. In this example, 20% of positive samples and 2% of negative samples were drawn from a population with elevated biomarker expression $\mathcal{N}(9,1)$ and all other samples were drawn from a population with unaltered biomarker expression $\mathcal{N}(6,1)$. Restriction of the dataset defines two subsets of samples - explicitly, marker^{HIGH} (orange) and marker^{LOW} (blue) samples. (b) The complete ROC curve is marked at the current restriction (red lines). Restricting the parts of the ROC curve corresponding to marker^{HIGH} (orange) or marker^{LOW} (blue) samples gives us restricted ROC curves, for which a restricted AUC (rAUC) can be calculated. This is equivalent to rescaling the respective part of the ROC curve. We show the rounded two-way partial area under the complete ROC curve for marker^{HIGH} (orange) or marker^{LOW} (blue) samples. Additionally, we show the rounded scaling factors corresponding to the reciprocal of the blue or orange rectangle area. The rAUC is then the two-way partial AUC multiplied with s. (c) The distribution of marker^{HIGH} samples from positive and negative samples in the orange rectangle from (a) and (b). (d) ROC curve calculated only on marker^{HIGH} samples. The complete AUC on only these marker^{HIGH} samples is shown top-left in black and is identical to the orange rescaled rAUC from (b). (e) The distribution of marker^{LOW} samples from positive and negative samples in the blue rectangle from (a) and (b). (f) ROC curve calculated only on marker^{LOW} samples. The complete AUC on only these marker^{LOW} samples is shown top-left in black and is identical to the blue rescaled rAUC from (b).

Supplementary Movie 4: Asinh transformed and rescaled gating for all 48 samples (GIF).

Following a conventional workflow, an experienced, blinded operator performed sample-wise manual recompensation of flow cytometry data using Kaluza software. Cell antigen-wise asinh-transformations and rescaling were then applied. Here, we illustrate our data preprocessing with 48 samples, which represented 6 replicate stainings from 8 healthy donors. (a) Shows the gating of all cells from one donor's replicate after cell antigen-wise asinh transformation. (b) Shows the gating of all cells from the same donor's replicate after cell antigen-wise asinh transformation and rescaling.
