# Supplementary figures and images for: Restricting datasets to classifiable samples augments discovery of immune disease biomarkers
Source: Nat Commun. 2024 Jun 26;15:5417. doi: 10.1038/s41467-024-49094-3 (PMC11208602; doi:10.1038/s41467-024-49094-3)

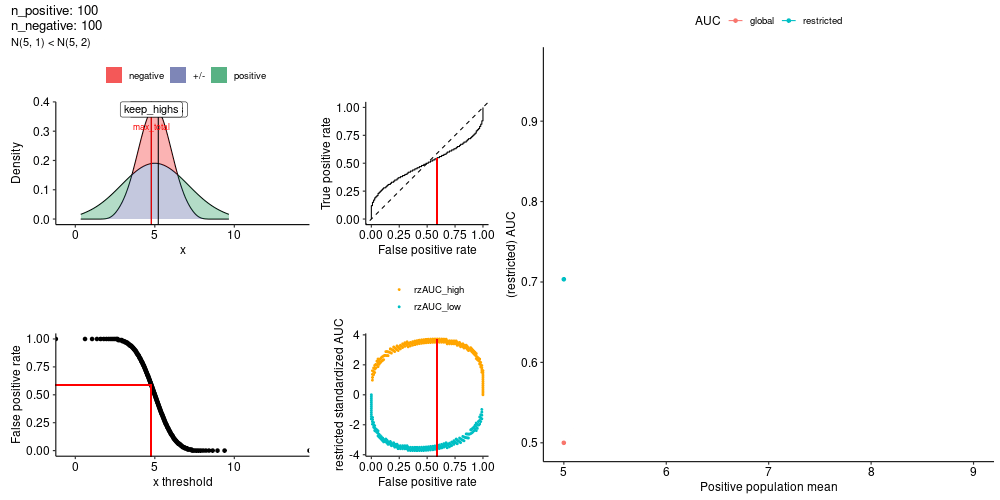

Supplement: Supplementary file 4 — Supplementary Movie 1 [file 41467_2024_49094_MOESM4_ESM.gif]

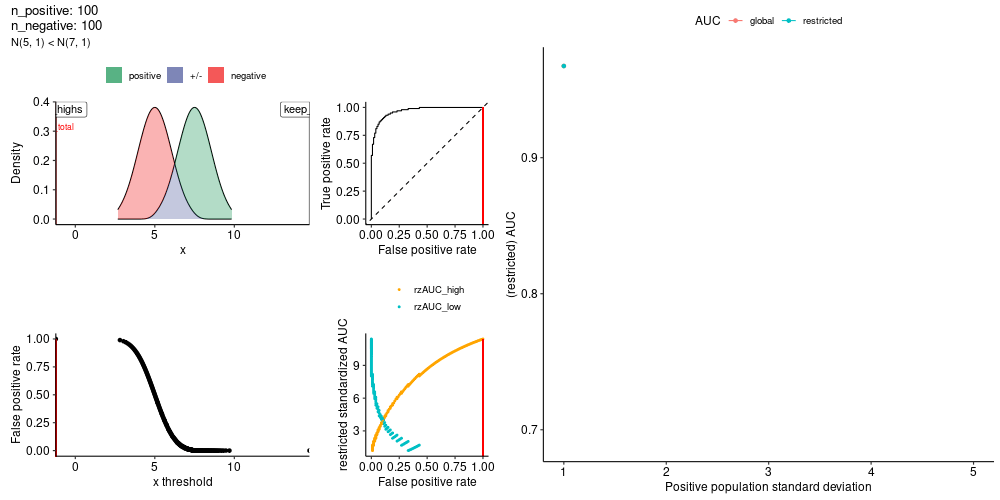

Supplement: Supplementary file 5 — Supplementary Movie 2 [file 41467_2024_49094_MOESM5_ESM.gif]

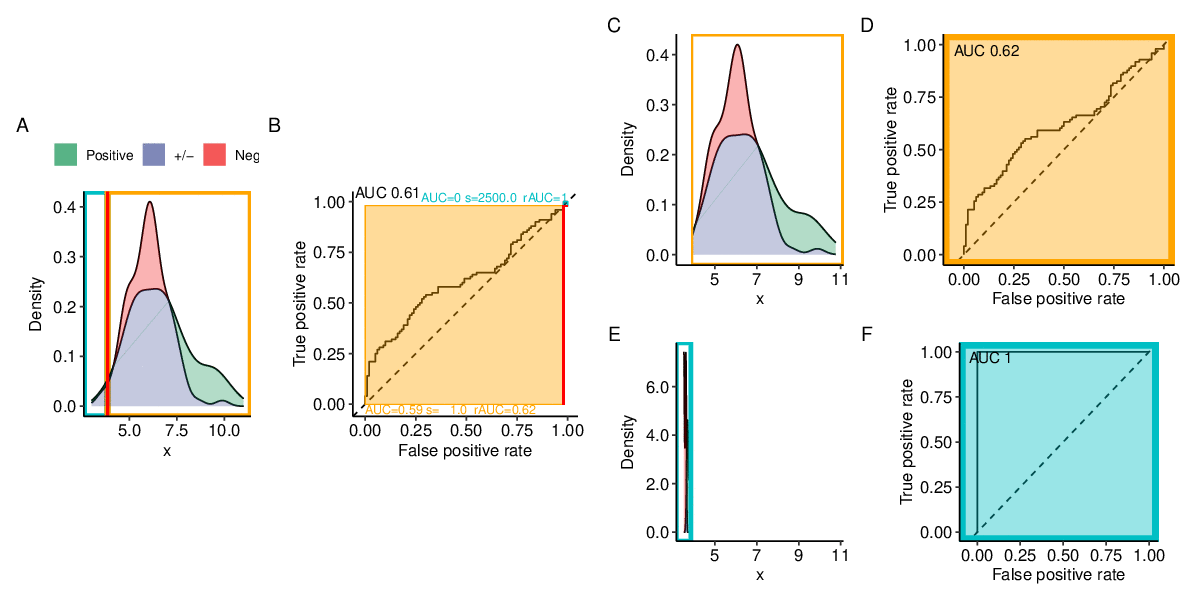

Supplement: Supplementary file 6 — Supplementary Movie 3 [file 41467_2024_49094_MOESM6_ESM.gif]

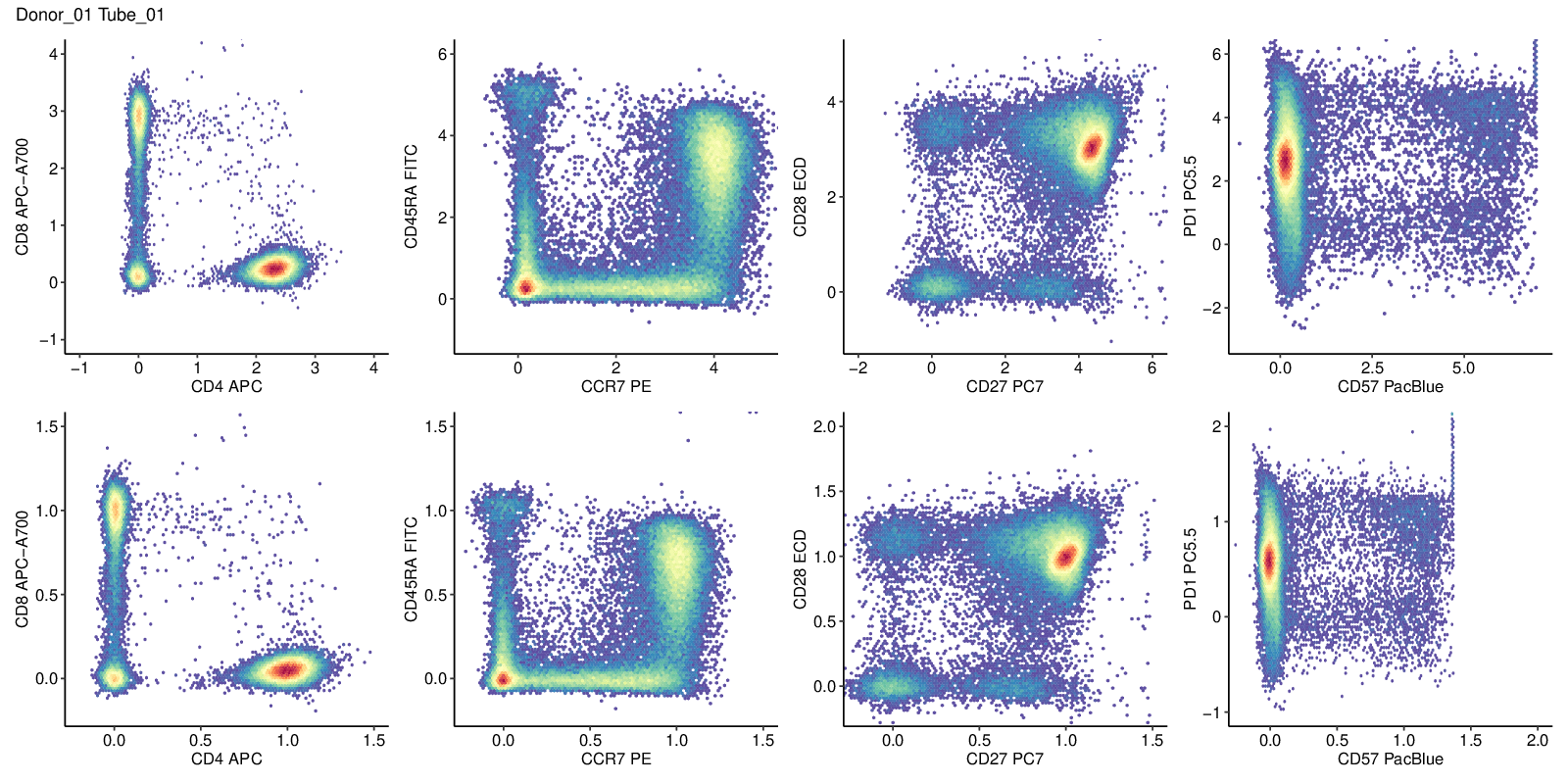

Supplement: Supplementary file 7 — Supplementary Movie 4 [file 41467_2024_49094_MOESM7_ESM.gif]
