# Supplementary material for: Restricting datasets to classifiable samples augments discovery of immune disease biomarkers
Source: Nat Commun. 2024 Jun 26;15:5417. doi: 10.1038/s41467-024-49094-3 (PMC11208602; doi:10.1038/s41467-024-49094-3)
Supplement: Supplementary file 8 — Reporting Summary [file 41467_2024_49094_MOESM8_ESM.pdf]

nature portfolio

Corresponding author(s): HUTCHINSON, James A.

Last updated by author(s): Feb 21, 2024

Reporting Summary

Nature Portfolio wishes to improve the reproducibility of the work that we publish. This form provides structure for consistency and transparency in reporting. For further information on Nature Portfolio policies, see our Editorial Policies and the Editorial Policy Checklist.

Statistics

For all statistical analyses, confirm that the following items are present in the figure legend, table legend, main text, or Methods section.

n/a	Confirmed	
☐	☒	The exact sample size (*n*) for each experimental group/condition, given as a discrete number and unit of measurement
☐	☒	A statement on whether measurements were taken from distinct samples or whether the same sample was measured repeatedly
☐	☒	The statistical test(s) used AND whether they are one- or two-sided<br>*Only common tests should be described solely by name; describe more complex techniques in the Methods section.*
☐	☒	A description of all covariates tested
☐	☒	A description of any assumptions or corrections, such as tests of normality and adjustment for multiple comparisons
☐	☒	A full description of the statistical parameters including central tendency (e.g. means) or other basic estimates (e.g. regression coefficient) AND variation (e.g. standard deviation) or associated estimates of uncertainty (e.g. confidence intervals)
☐	☒	For null hypothesis testing, the test statistic (e.g. *F*, *t*, *r*) with confidence intervals, effect sizes, degrees of freedom and *P* value noted<br>*Give P values as exact values whenever suitable.*
☒	☐	For Bayesian analysis, information on the choice of priors and Markov chain Monte Carlo settings
☒	☐	For hierarchical and complex designs, identification of the appropriate level for tests and full reporting of outcomes
☒	☐	Estimates of effect sizes (e.g. Cohen's *d*, Pearson's *r*), indicating how they were calculated

Our web collection on statistics for biologists contains articles on many of the points above.

Software and code

Policy information about availability of computer code

Data collection	Cytometry List Mode Data Acquistion and Analysis Software Version 1.3 (Beckman Coulter) commercial software
Data analysis	The full analysis code can be found on figshare (FIGSHARE LINK HERE)<br><br>Kaluza version 2.1 (Beckman Coulter) commercial software<br><br>Developed packages:<br>dataMelanoma - 0.0.7 - (R) (https://github.com/ggrlab/dataMelanoma DOI: 10.5281/zenodo.10718835<br>restrictedROC - 3.3.3 - (R) https://github.com/ggrlab/restrictedROC DOI: 10.5281/zenodo.10718838<br>nbnode - v1.1.0 - (Python) https://github.com/ggrlab/nbnode DOI: 10.5281/zenodo.10718837<br><br>R (See also print(sessionInfo()) in supplied code.)<br><br>R 4.3.2<br>xtable - 1.8-4<br>tablesgg - 0.8-1<br>tibble - 3.2.1<br>gifski - 1.12.0-2<br>magick - 2.8.1<br>gganimate - 1.0.8

ggforce - 0.4.1
ggpointdensity - 0.1.0
tidyr - 1.3.0
patchwork - 1.1.3
future - 1.33.0
dplyr - 1.1.3
ggcyto - 1.28.1
flowWorkspace - 4.12.2
ncdfFlow - 2.46.0
BH - 1.81.0-1
ggpubr - 0.6.0
ggplot2 - 3.4.4
flowCore - 2.12.2
pacman - 0.5.1

Dependencies:
gridExtra_2.3, remotes_2.4.2.1, rlang_1.1.1, magrittr_2.0.3, matrixStats_1.0.0, compiler_4.3.2, callr_3.7.3, vctrs_0.6.4, stringr_1.5.0, profvis_0.3.8, pkgconfig_2.0.3, crayon_1.5.2, fastmap_1.1.1, backports_1.4.1, ellipsis_0.3.2, utf8_1.2.4, promises_1.2.1, sessioninfo_1.2.2, graph_1.78.0, ps_1.7.5, purrr_1.0.2, xfun_0.41, zlibbioc_1.46.0, cachem_1.0.8, progress_1.2.2, later_1.3.1, tweenr_2.0.2, broom_1.0.5, parallel_4.3.2, prettyunits_1.2.0, R6_2.5.1, tables_0.9.17, stringi_1.7.12, RColorBrewer_1.1-3, parallelly_1.36.0, car_3.1-2, pkgload_1.3.3, knitr_1.45, Rcpp_1.0.11, usethis_2.2.2, httpuv_1.6.12, tidyselect_1.2.0, abind_1.4-5, codetools_0.2-19, miniUI_0.1.1.1, processx_3.8.2, listenv_0.9.0, pkgbuild_1.4.2, lattice_0.22-5, plyr_1.8.9, Biobase_2.60.0, shiny_1.7.5.1, withr_2.5.2, desc_1.4.2, urlchecker_1.0.1, polyclip_1.10-6
, pillar_1.9.0, carData_3.0-5, stats4_4.3.2, generics_0.1.3, rprojroot_2.0.3, hms_1.1.3, S4Vectors_0.38.2, munsell_0.5.0, scales_1.2.1, globals_0.16.2, glue_1.6.2, tools_4.3.2, hexbin_1.28.3, data.table_1.14.8, ggsignif_0.6.4, fs_1.6.3, XML_3.99-0.15, grid_4.3.2, RProtoBufLib_2.12.1, devtools_2.4.5, colorspace_2.1-0, cli_3.6.1, fansi_1.0.5, cytolib_2.12.1, Rgraphviz_2.44.0, gtable_0.3.4, rstatix_0.7.2, digest_0.6.33, BiocGenerics_0.46.0, htmlwidgets_1.6.2, farver_2.1.1, memoise_2.0.1, htmltools_0.5.6.1, lifecycle_1.0.3, mime_0.12, MASS_7.3-60

Python (See $CONDA_PREFIX/bin/pip freeze in supplied code)

python - 3.8.12
helping-analysis @ file:///home/gugl/clonedgit/paper/2023-Glehr_rROC_code/packages/helping_analysis

Dependencies:
anytree==2.8.0, black==22.3.0, certifi==2022.12.7, charset-normalizer==3.1.0, click==8.1.3, colour==0.1.5, contourpy==1.0.7, cycler==0.11.0, datatable==1.0.0, dirichlet==0.9, docopt==0.6.2, dtreeviz==2.2.1, exceptiongroup==1.1.1, fonttools==4.39.3, gitdb==4.0.9, gitlabber==1.1.9, GitPython==3.1.27, globre==0.1.5, graphviz==0.20.1, idna==3.4, importlib-resources==5.12.0, iniconfig==2.0.0, Jinja2==3.1.2, joblib==1.2.0, kiwisolver==1.4.4, MarkupSafe==2.1.2, matplotlib==3.7.1, mypy-extensions==0.4.3, numpy==1.24.3, packaging==23.1, pandas==2.0.1, pathspec==0.9.0, Pillow==9.5.0, pluggy==1.0.0, psutil==5.9.5, pyarrow==11.0.0, pydotplus==2.0.2, pyparsing==3.0.9, pytest==7.3.1, python-dateutil==2.8.2, python-gitlab==3.3.0, pytz==2023.3, requests==2.29.0, requests-toolbelt==0.9.1, scikit-learn==1.2.2, scipy==1.10.1, six==1.16.0, smmap==5.0.0, threadpoolctl==3.1.0, tomli==2.0.1, torch==1.12.1+cpu, torch-geometric==2.3.1, torch-scatter==2.1.0, torch-sparse==0.6.16, tqdm==4.65.0, typing_extensions==4.5.0, tzdata==2023.3, urllib3==1.26.15, zipp==3.15.0

For manuscripts utilizing custom algorithms or software that are central to the research but not yet described in published literature, software must be made available to editors and reviewers. We strongly encourage code deposition in a community repository (e.g. GitHub). See the Nature Portfolio guidelines for submitting code & software for further information.

Data

Policy information about availability of data

All manuscripts must include a data availability statement. This statement should provide the following information, where applicable:

- Accession codes, unique identifiers, or web links for publicly available datasets
- A description of any restrictions on data availability
- For clinical datasets or third party data, please ensure that the statement adheres to our policy

The authors declare that all data supporting the findings of this study are available within the paper, its supplementary information files and downloadable files deposited at figshare (https://doi.org/10.6084/m9.figshare.22759076).

Research involving human participants, their data, or biological material

Policy information about studies with human participants or human data. See also policy information about sex, gender (identity/presentation), and sexual orientation and race, ethnicity and racism.

Reporting on sex and gender	The sex and age distribution of patients enrolled in the Training and Validation sets is reported in Supplementary Table 3. Healthy volunteers taken as a training cohort for our synthetic flow cytometry data were selected to achieve an equal balance of male and females. This manuscript does not report the gender of healthy donors or patients.
Reporting on race, ethnicity, or	We report that all healthy volunteers and patients were sampled from local populations - either regular thrombocyte donors or patients attending the Dermatology Outpatient Clinic to receive treatment for advanced melanoma. Consequently, most

other socially relevant groupings	patients were white caucasians. This manuscript does not explicitly report information about race, ethnicity or other socially relevant groupings. No stratification of data was made according to these criteria.
Population characteristics	Patients enrolled in the training and validation sets had a diagnosis of stage III or IV melanoma. All patients were already scheduled to receive combined Ipilimumab plus Nivolumab therapy at the time of recruitment. A complete clinical summary of patient characteristics is given as Supplementary Table 3, including baseline staging, previous therapies, Ipi-Nivo cycles and incidence of immune-related adverse reactions. In the training set of n=110: male = 66.4%; female = 33.6%; mean age = 62 y; mean BMI = 26.6; liver metastases were present in 27.3% cases. In the validation set of n=30: male = 60.0%; female = 40.0%; mean age = 64 y; mean BMI = 28.3; liver metastases were present in 30.0% cases.
Recruitment	Adult patients attending the UKR Dermatology Outpatient Clinic were recruited sequentially to a non-randomized, single arm, observational study. Enrollment required that patients provided informed, written consent to study participation. According to clinical outcomes after treatment, patients were split into affected and unaffected sets. Predictive models were trained on pretreatment measurements; therefore, we see no selection or other bias. Predictive models were then prospectively validated using sequentially collected cases.
Ethics oversight	University of Regensburg Ethics Committee. (1) Training and validation sets - approval 16-101-0125. (2) Healthy volunteers - approval 22-2780-01.

Note that full information on the approval of the study protocol must also be provided in the manuscript.

Field-specific reporting

Please select the one below that is the best fit for your research. If you are not sure, read the appropriate sections before making your selection.

☒ Life sciences ☐ Behavioural & social sciences ☐ Ecological, evolutionary & environmental sciences

For a reference copy of the document with all sections, see nature.com/documents/nr-reporting-summary-flat.pdf

Life sciences study design

All studies must disclose on these points even when the disclosure is negative.

Sample size	The training set of n=110 patients was previously published as a data resource by our group (Glehr et al. 2022. Front Immunol.) To estimate the required size of the prospective validation set, we performed a univariate power calculation using Fisher's Exact test, assuming α=0.05, 1-β=0.80 and an effect size estimate based upon PPV=0.8 and NPV=0.8.
Data exclusions	No data were excluded. To build and validate random forests, a marker-wise filtering of restricted datasets was performed to remove all markers with informative values in fewer than 10 % samples - please see Figure 8.
Replication	We present a multivariate model trained on n=110 cases. This was validated using a prospectively collected set of n=30 patients.
Randomization	The training set and prospective validation set were populated sequentially. Training set patients were recruited between Oct 2016 and Jun 2021. A predictive model was then established and a prospective validation study was conducted. Validation set patients were recruited between Jun 2021 and Jan 2023. Sequential and separate recruitment of training and validation sets is a gold-standard approach in biomarker discovery.
Blinding	This was a single-arm observational study. Blood samples were drawn from patients before starting therapy. The clinicians responsible for diagnosing treatment-related complications were blind to the immunological data and output of our predictive models.

Reporting for specific materials, systems and methods

We require information from authors about some types of materials, experimental systems and methods used in many studies. Here, indicate whether each material, system or method listed is relevant to your study. If you are not sure if a list item applies to your research, read the appropriate section before selecting a response.

Materials & experimental systems

n/a	Involved in the study	
☐	☒	Antibodies
☒	☐	Eukaryotic cell lines
☒	☐	Palaeontology and archaeology
☒	☐	Animals and other organisms
☐	☒	Clinical data
☒	☐	Dual use research of concern
☒	☐	Plants

Methods

n/a	Involved in the study	
☒	☐	ChIP-seq
☐	☒	Flow cytometry
☒	☐	MRI-based neuroimaging

Antibodies

Antibodies used	Throughout the manuscript, we report flow cytometry data generated with a highly validated clinical research panel, the DuraClone IM T cell Subsets Tube (Beckman Coulter, B53328). This panel incorporates the following CE-labelled, fluorochrome-conjugated mAb at predetermined concentrations: CD45RA-FITC, clone 2H4; CCR7-PE, clone G043H7; CD28-ECD, clone CD28.2; PD1-PC5.5, clone PD1.3; CD27-PC7, clone 1A4CD27; CD4-APC, clone 13B8.2; CD8-A700, clone B9.11; CD3-APC-A750, clone UCHT1; CD57-PB, clone NC1; CD45-KrO, clone J33.<br><br>Supplementary Figure 2 presents the analysis of human innate lymphocytes from patients with advanced melanoma using a 13-colour flow cytometry panel, as follows: TCR Vα7.2 (clone 3C10, mIgG1, AF488, 3 µl/reaction, BioLegend, 351703); IL-17 (clone BL168, PE, mIgG1, AF488, 2 µl/reaction, BioLegend, 512306); CD8 (clone RPA-T8, mIgG1, AF594, 3 µl/reaction, BioLegend, 301056); TCRγδ (clone IMMU510, mIgG1, PC5.5, 6 µl/reaction, Beckman Coulter, A98021); TCR Vα24 (clone 6B11, mIgG1, PC7, 3 µl/reaction, BioLegend, 342911); IL-4 (clone MPA-25D2, rIgG1, APC, 1 µl/reaction, BioLegend, 500812); CD4 (clone SK3, mIgG1, AF700, 3 µl/reaction, BioLegend, 344621); CD3 (clone UCHT1, mIgG1, APC-A750, 5 µl/reaction, Beckman Coulter, A94680); IFN-γ (clone B27, mIgG1, BV421, 2 µl/reaction, BioLegend, 506538); TNF-α (clone Mab11, mIgG1, BV510, 2 µl/reaction, BioLegend, 502950); CD69 (clone FN50, mIgG1, BV605, 5 µl/reaction, BioLegend, 310937); CD56 (clone 5.1H11, mIgG1, BV785, 5 µl/reaction, BioLegend, 362549); ViaKrome-818 viability dye (2 µl/reaction, Beckman Coulter, C36628).
Validation	The manufacturer supplies lot-by-lot certificates of analysis (see https://www.beckman.de/search#q=B53328&t=coveo-tabtechdocs). Please also see previous publications from our group: Hutchinson et al. (2021) Nat. Comms; Glehr et al. (2022) Front Immunol.

Clinical data

Policy information about clinical studies

All manuscripts should comply with the ICMJE guidelines for publication of clinical research and a completed CONSORT checklist must be included with all submissions.

Clinical trial registration	clinicaltrials.gov: NCT04158544
Study protocol	The full study protocol in German is available upon request from the authors.
Data collection	Patients were enrolled during outpatient assessment at the UKR Dermatology Clinic. Blood samples were drawn immediately prior to administration of the first dose of Ipi-Nivo therapy in the Comprehensive Cancer Center treatment suites at UKR.
Outcomes	The primary clinical outcomes were incidence of immunotherapy-related hepatitis or colitis. Clinical response was recorded as a secondary clinical outcome. All irAE were evaluated by an expert Dermatological Oncologist. ICI-related hepatitis was diagnosed when: (i) GOT, GPT, γ-GT or total bilirubin substantially deviated from pretreatment values; (ii) this change was not attributable to other causes, such as co-medication or viral disease; and (iii) liver injury was sufficiently severe that ICI therapy was suspended or stopped, or immunosuppression was started. Colitis was diagnosed when increased stool frequency or loose consistency, accompanied by abdominal discomfort led to suspension or cessation of ICI therapy and introduction of immunosuppressive treatment. Clinical responses were assessed using the Response Evaluation Criteria in Solid Tumors (RECIST 1.1). Patients with progressive disease were categorized as non-responders, whereas those with complete or partial responses, and those with stable disease, were categorized as responders.

Plants

Seed stocks	No plants were used in this study
Novel plant genotypes	No plants were used in this study
Authentication	No plants were used in this study

Flow Cytometry

Plots

Confirm that:

☒ The axis labels state the marker and fluorochrome used (e.g. CD4-FITC).

☒ The axis scales are clearly visible. Include numbers along axes only for bottom left plot of group (a 'group' is an analysis of identical markers).

☒ All plots are contour plots with outliers or pseudocolor plots.

☒ A numerical value for number of cells or percentage (with statistics) is provided.

Methodology

Sample preparation	Step-by-step protocols for preparing and analyzing clinical samples by flow cytometry can be accessed through Protocol Exchange. Briefly, blood was collected into EDTA-vacutainers by peripheral venepuncture then delivered to the responsible lab at ambient temperature. Samples were stored at 4°C for up to 4 h before processing. Whole blood samples were stained using the DURAClone IM T Cell Subsets Tube (Beckman Coulter, B53328).
Instrument	(1) Navios, Beckman Coulter; (2) Navios EX, Beckman Coulter; (3) CytoFlex LX, Beckman Coulter (Fig.S2).
Software	Navios: Cytometry List Mode Data Acquisition and Analysis Software version 1.3 (Beckman Coulter).
Cell population abundance	Cells were not sorted.
Gating strategy	An experienced operator performed blinded analyses following a conventional workflow that entailed sample-wise recompensation, arcsinh transformation and rescaling before applying a uniform gating strategy (Supplementary Fig 4). Leucocytes were gated using biaxial plots of SSC vs CD45. Singlets were gated using biaxial plots of FSC vs FS-TOF. CD3+ T cells were gated using biaxial plots of FSC vs CD3. All subsequent analyses were performed using computational routines developed and described in this article.

☒ Tick this box to confirm that a figure exemplifying the gating strategy is provided in the Supplementary Information.
